# Graded functional organization in the left inferior frontal gyrus: evidence from task-free and task-based functional connectivity

**DOI:** 10.1093/cercor/bhad373

**Published:** 2023-10-13

**Authors:** Veronica Diveica, Michael C Riedel, Taylor Salo, Angela R Laird, Rebecca L Jackson, Richard J Binney

**Affiliations:** Department of Psychology & Cognitive Neuroscience Institute, Bangor University, Bangor, Wales LL57 2AS, United Kingdom; Department of Neurology and Neurosurgery & Montreal Neurological Institute, McGill University, Montreal, QC H3A 2B4, Canada; Department of Physics, Florida International University, Miami, FL 33199, United States; Department of Psychiatry, University of Pennsylvania, Philadelphia, PA, 19104, United States; Department of Physics, Florida International University, Miami, FL 33199, United States; Department of Psychology & York Biomedical Research Institute, University of York, York, YO10 5DD, United Kingdom; Department of Psychology & Cognitive Neuroscience Institute, Bangor University, Bangor, Wales LL57 2AS, United Kingdom

**Keywords:** ventrolateral prefrontal cortex, connectivity-based parcellation, meta-analysis, cognitive control, cross-domain

## Abstract

The left inferior frontal gyrus has been ascribed key roles in numerous cognitive domains, such as language and executive function. However, its functional organization is unclear. Possibilities include a singular domain-general function, or multiple functions that can be mapped onto distinct subregions. Furthermore, spatial transition in function may be either abrupt or graded. The present study explored the topographical organization of the left inferior frontal gyrus using a bimodal data-driven approach. We extracted functional connectivity gradients from (i) resting-state fMRI time-series and (ii) coactivation patterns derived meta-analytically from heterogenous sets of task data. We then sought to characterize the functional connectivity differences underpinning these gradients with seed-based resting-state functional connectivity, meta-analytic coactivation modeling and functional decoding analyses. Both analytic approaches converged on graded functional connectivity changes along 2 main organizational axes. An anterior–posterior gradient shifted from being preferentially associated with high-level control networks (anterior functional connectivity) to being more tightly coupled with perceptually driven networks (posterior). A second dorsal–ventral axis was characterized by higher connectivity with domain-general control networks on one hand (dorsal functional connectivity), and with the semantic network, on the other (ventral). These results provide novel insights into an overarching graded functional organization of the functional connectivity that explains its role in multiple cognitive domains.

## Introduction

The left inferior frontal gyrus (LIFG) is ascribed a key role in numerous cognitive domains, including language (Friederici 2011), semantics (Lambon Ralph et al. 2017), action (Papitto et al. 2020), social cognition (Diveica et al. 2021), and executive function (Fedorenko et al. 2013). The extent of this overlap is remarkable, but what is driving it is unknown. One possibility is that the LIFG subserves a singular function that manifests as common activation across domains. Alternatively, a detailed exploration of its organization could reveal subregions with multiple functional specializations.

Some clues are gleaned from studies of cellular microstructure and white-matter connectivity that date back to Brodmann (1909). Cytoarchitecture and “fibrillo-architecture” are proposed to determine a region’s functional characteristics by constraining local processing capabilities and the incoming/outgoing flow of information, respectively (Passingham et al. 2002; Cloutman and Lambon Ralph 2012). Indeed, these data reveal that the LIFG is far from uniform and, instead, comprises at least 3 subregions with distinct cytoarchitecture (Amunts et al. 1999; Schenker et al. 2008; Wojtasik et al. 2020), neurotransmitter receptor distributions (Amunts et al. 2010), and structural connectivity (Anwander et al. 2007; Klein et al. 2007; Neubert et al. 2014; Wang et al. 2020). However, the topographic relationship between structural divisions and functional specialization has thus far proven difficult to solve.

Various functional dissociations have been identified within the LIFG by means of functional neuroimaging, including, for example, distinctions between domain-specific (e.g. language-related) and task-general cognitive systems (Fedorenko et al. 2012; Hodgson et al. 2021), between semantic and phonological processing (Devlin et al. 2003; Hodgson et al. 2021), and between memory retrieval and postretrieval selection mechanisms (Badre et al. 2005). It is not yet clear, however, how these observations fit together and whether they can be reconciled under a single unifying framework. One possibility is that there are multiple axes along which LIFG function dissociates. For instance, some functional dissociations are characterized as transitioning along an anterior to posterior axis of the LIFG (e.g. Badre and Wagner 2007; Gough et al. 2005; Spunt and Lieberman 2012), while others also comprise a dorsal–ventral distinction (e.g. Fedorenko et al. 2012; Hodgson et al. 2021; Poldrack et al. 1999). Moreover, the manner in which these transitions occur is also not clear; there may be sharp borders that delineate LIFG subregions (Fedorenko et al. 2012; Fedorenko and Blank 2020), but this contrasts with claims that there are gradual changes in function and areas of overlap (e.g. Choi et al. 2018; Devlin et al. 2003). One of the key reasons that it is challenging to draw together various functional dissociations is that they have been studied in isolation from each other and primarily via hypothesis-driven approaches to small *N* datasets. This can be overcome by investigating LIFG function via a large-scale data-driven approach and applying it to a dataset that spans multiple cognitive domains (Genon et al. 2018). This approach could better capture the full functional repertoire of a brain region and thereby tease out the underlying and generalizable organizational principles.

Functional connectivity (FC) patterns derived from neuroimaging data are particularly well suited for this aim as they are able to capture the extent to which regional activation covaries over time and, therefore, are sensitive to context-dependent inter-regional interactions. Moreover, they can reveal aspects of the connectome that might not manifest within other modalities (e.g. anatomically derived connectivity); FC can arise between anatomically remote brain areas without direct structural connections (Damoiseaux and Greicius 2009; Suárez et al. 2020). The small number of studies that have attempted to use FC to divide the LIFG into subregions reveal a heterogenous functional architecture (Kelly et al. 2010; Clos et al. 2013). However, they are limited by the fact that they implemented “hard” clustering algorithms, which assume that sharp borders separate intrinsically homogeneous neural regions (Eickhoff et al. 2015). This means they may fail to identify graded transitions that (i) could give rise to functionally intermediate areas (Bailey and Von Bonin 1951; Rosa and Tweedale 2005) and (ii) have been observed in the connectivity patterns of other brain regions (e.g. Cerliani et al. 2012; Bajada et al. 2017; Jackson et al. 2018, 2020; Tian and Zalesky 2018), as well as within the cytoarchitecture of the transmodal cortex (Brodmann 1909; Bailey and Von Bonin 1951). Therefore, the possibility of graded functional differences in the LIFG remains unexplored.

In systems neuroscience, there is growing interest in the potential to advance understanding of brain structural and functional organization using an emergent analytical approach that is designed to capture cortical *gradients* (Huntenburg et al. 2018; Bajada et al. 2020; Bernhardt et al. 2022). Cortical gradients can be defined as continuous spatial transitions in neural features, and they can capture overlapping organizing axes of the cerebral cortex. As such, gradients are well suited for exploring the organization of brain regions that exhibit connection topographies that imply both functional heterogeneity and functional multiplicity (i.e. overlapping functional modes; Haak and Beckmann 2020). Practically speaking, gradient analysis is a dimensionality reduction technique that identifies the spatial ordering of regions/voxels in a lower-dimensional yet continuous space based on similarity of features (for more detailed descriptions, see Bajada et al. 2020; Haak et al. 2018; Vos de Wael et al. 2020). They produce “gradient maps” that describe how much brain voxels/areas resemble each other in terms of cytoarchitecture, or structural/functional connections, for example, and provide a spatial framework for interpreting and linking functions across different task domains.

In seminal work, Margulies et al. (2016) used gradient analysis to examine variation in brain-wide (task-free) FC and revealed the presence of a “principal functional gradient”. At one end of the gradient lie unimodal systems that underpin sensory and motor function and, at the other end, lie hetero- and supra-modal association cortices. This macroscale property of cortical organization is thought to have an important functional role, which is untethering of the heteromodal cortex from sensory constraints (Mesulam 1998; Buckner and Krienen 2013) and driving cross-modal integration, which ultimately gives rise to more abstract cognitive functions (Huntenburg et al. 2018; Smallwood et al. 2021). At the level of this principal macroscale gradient, the LIFG resembles heteromodal brain regions. Moreover, the FC patterning within the LIFG appears largely homogeneous. However, gradient analysis has the potential to reveal more fine-grained functional distinctions when applied to restricted volumes of interest, as opposed to the whole brain (Haak and Beckmann 2020). Yet, to date, this potential remains unexplored with respect to the LIFG.

As mentioned above, the few previous attempts to parcellate the LIFG using FC have utilized “hard” clustering algorithms, and gradient analyses have a number of advantages over these more standard brain parcellation techniques. First, a key feature is that they do not presuppose the nature of variation and, therefore, can be used to demonstrate both graded changes and discrete boundaries (Johansen-Berg et al. 2004; Bajada et al. 2017; Jackson et al. 2018). Moreover, gradient analyses can be used as a data-driven means to probe the *degree of gradation* in cortical organization (Bajada et al. 2020). Second, gradient analyses can identify and distinguish between superimposed but orthogonal dimensions of functional variation. In the context of other parcellation techniques, these overlapping functional modes could appear as a singular component of organization (Haak et al. 2018). This is important because brain regions can exhibit multiple coexisting trajectories of functional change (for an extensive discussion, see Haak and Beckmann 2020). For example, the primary visual cortex represents distance from the center of the retina along the calcarine sulcus, whereas the angle is represented perpendicular to this (Wandell et al. 2005, 2007). The existence of multiple superimposed modes of functional change in the LIFG might explain why previous functional neuroimaging studies have identified functional differences along different spatial dimensions. Finally, a presence of overlapping and graded connection topographies would have implications for theories of LIFG function in terms of the computational complexity afforded to it (Jbabdi et al. 2013). For example, Haak and Beckmann (2020) suggest that this is an efficient way to wire a system and that it allows the implementation of transformations between and across different representational systems.

To summarize, the objectives of the current study were as follows. We sought to reconcile various functional dissociations that have been identified within the LIFG by taking a data-driven approach to exploring variation in FC that spans a broad set of cognitive states/tasks. We applied a gradient analysis to LIFG FC for the first time so that it would be possible to (i) detect multiple superimposed spatial dimensions of organization and (ii) identify both discrete changes in function and graded changes that could give rise to intermediate areas and functional multiplicity. Thereby, we aimed to elucidate an overarching spatial framework that explains the LIFG’s role in multiple cognitive domains.

## Materials and methods

**Fig. 1:**
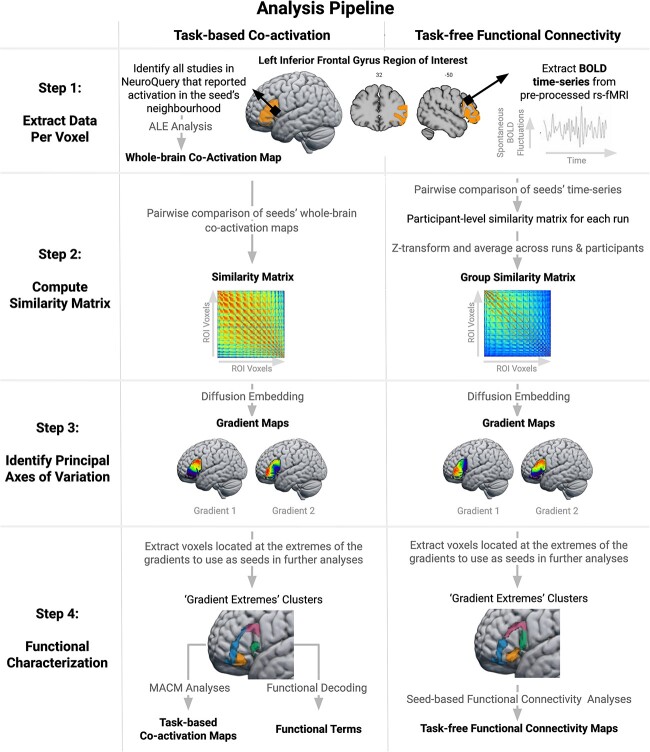
Schematic overview of the analytic pipeline. The output of each analysis step is highlighted in bold. In the first step, we estimated the whole-brain coactivation patterns of individual ROI voxels using meta-analytic coactivation modeling (first column) and extracted their resting-state BOLD time-series (second column). Then, we performed a pairwise comparison of each voxel’s coactivation patterns/time-series using the product-moment correlation coefficient. This resulted in 2 similarity matrices, which were subsequently used as input for gradient analyses (diffusion embedding algorithm) in order to identify the main axes of variation across the ROI. In the final step, we performed MACM, functional decoding and seed-based resting-state FC analyses on “gradient extremes” clusters extracted from the opposite ends of the gradient maps in order to identify the FC patterns between which FC varies in the LIFG. The code used for data analysis can be accessed at: osf.io/u2834/.

We used a data-driven approach to explore LIFG gradients based on 2 measures of FC: (i) correlations in task-free fMRI time-series and (ii) meta-analytically derived patterns of task-driven coactivation from across multiple cognitive domains. This bimodal approach not only allowed us to validate our results using independent datasets but also made it possible to assess the generalizability of the functional organization of the LIFG across different mental states. Indeed, one data type captures activation patterns associated with spontaneous thought (e.g. a state of mind-wandering; Chou et al. 2017; Doucet et al. 2012), while the other is assumed to reflect mental processes constrained by extrinsic demands (Laird et al. 2013). The summary of our analytical approach is as follows. For each voxel, we (i) extracted BOLD fluctuations over time from resting-state fMRI scans and (ii) meta-analytically identified the brain voxels with which it consistently coactivates across a broad range of task demands. Then, for each FC modality, we compared the fMRI time-series/coactivation patterns of each pair of voxels within the LIFG region of interest (ROI; see sections Task-Free FC Similarity Matrix and Task-Based Coactivation Similarity Matrix). We conducted gradient analyses on the resulting similarity matrices to extract the principal axes of variation and to estimate the degree to which there are discrete or graded changes in function (see section Gradient Analysis). In a second step, we conducted descriptive analyses to understand which FC differences gave rise to these gradients (see section Functional Characterization). To this end, we performed seed-based resting-state FC and meta-analytic coactivation modeling (MACM) analyses on clusters extracted from the extreme ends of the identified gradients (henceforth “gradient extremes” clusters). Finally, we probed the functional/task terms (e.g. “cognitive control,” “language”) associated with these IFG subregions using functional decoding analyses. A schematic overview of the analytic pipeline is illustrated in Fig. 1.

### Definition of the LIFG region of interest

The LIFG ROI was created by combining the pars opercularis, pars triangularis, and pars orbitalis as delineated in the second release of the Automated Anatomical Labeling (AAL2) atlas (Rolls et al. 2015). In addition, we included the region termed lateral orbital gyrus in the AAL2 parcellation because it is considered to pertain to pars orbitalis (Keller et al. 2009). These regions correspond roughly to Brodmann areas 44, 45, and (part of) 47. We retained only the voxels with 50% or greater probability of being gray matter according to the ICBM-152 template (Fonov et al. 2011). To ensure that the ROI did not encompass regions within neighboring gyri that were of no interest to the present study, the ROI was manually cleaned by removing voxels that crossed gyral boundaries into the precentral gyrus and middle frontal gyrus in the MNI-152 T1 template included in FSL (version 6.0.1). The final ROI comprised 1,813 (2 × 2 mm) voxels and is depicted in Fig. 1 (Step 1) and available at: osf.io/u2834/.

### Data

#### Resting-state fMRI data

To assess the functional organization of the LIFG based on task-free FC, we used the resting-state fMRI time-series of 150 randomly selected healthy young adult participants (77 females) from the Human Connectome Project S1200 release (Van Essen et al. 2013). For each participant, data were available from up to 4 15-min runs of resting-state fMRI scans collected using the acquisition protocol described by Smith et al. (2013). All 4 scans were available for 139 participants (92.7% of participant sample), only 3 scans for 3 participants (2%), and only 2 scans for 8 participants (5.3%). The data were already preprocessed in the MNI space using the minimal processing pipeline described by Glasser et al. (2013) and de-noised using ICA-FIX (Salimi-Khorshidi et al. 2014). We regressed the global signal to further reduce the effects of motion artifacts (Burgess et al. 2016), and smoothed the images using a 4-mm full-width half-maximum Gaussian kernel. In keeping with other resting state studies, we took an additional step of band-pass filtering the data to retain only frequencies between 0﻿.01 and 0.08 Hz (Satterthwaite et al. 2013).

#### Meta-analytic functional neuroimaging data

To assess the functional organization of the LIFG based on task coactivation patterns, we adopted a meta-analytic approach and capitalized on the openly available NeuroQuery database (neuroquery.org). NeuroQuery contains over 400,000 activation coordinates that were automatically extracted from 13,459 neuroimaging studies (Dockès et al. 2020). The database also includes estimates of frequency of occurrence of 6,308 terms (e.g. “cognitive control,” “semantic memory”) in each full-text publication from this corpus, which were used to perform functional decoding (see section Functional Characterization).

### Data analysis

#### Task-free FC similarity matrix

To compute the task-free FC similarity matrix, we first extracted the blood-oxygen-level-dependent signal time-series of every voxel within the ROI, resulting in a voxel by timepoint matrix for each participant and each run. Then, we computed a cross-correlation matrix by calculating the product-moment correlation coefficient between the time-series of all pairs of ROI voxels. The resulting voxel by voxel matrix was *z*-score-normalized to allow the result of each run to be averaged (Dunlap et al. 2013), in order to generate an average similarity matrix across runs per participant. These participant-level matrices were subsequently averaged resulting in a group similarity matrix. This task-free FC-based similarity matrix was transformed back from *z*-scores to correlation values for gradient decomposition (see section Gradient Analysis).

#### Task-based coactivation similarity matrix

To compute the task-based coactivation similarity matrix, we first used MACM analyses to identify the brain areas consistently coactivated with each voxel within the ROI. MACM uses meta-analytic data to quantify the co-occurrence of activation between voxels across a broad range of task demands (Laird et al. 2013). This analysis involved extracting all studies in the NeuroQuery database that reported at least 1 activation peak within 6 mm of a given voxel. Next, we quantified the convergence of activation across the identified experiments using the revised activation likelihood estimation (ALE) algorithm (Eickhoff et al. 2012) as implemented in the Python library NiMARE (Salo et al. 2022). This process was repeated for all voxels within the ROI, resulting in 1,813 unthresholded MACM maps that estimate the strength of coactivation between each ROI voxel and all other brain voxels (ROI voxel by brain voxel matrix). In the second step, we generated a cross-correlation matrix by calculating the product-moment correlation coefficient between the MACM map values of each pair of ROI voxels. The resulting task-based coactivation similarity matrix was used as input for the gradient analysis (see section Gradient Analysis).

#### Gradient analysis

We conducted gradient analyses to separately explore the principal axes of variation in task-free FC and task-based coactivation patterns across the ROI. To this end, we first sparsified the similarity matrices by retaining only the top 10% of values row-wise and computed a symmetric affinity matrix using a cosine kernel. The application of this threshold ensures that the results are only based on strong connections, rather than weak and potentially spurious connections (Vos de Wael et al. 2020).

Then, we generated gradient maps by using the diffusion embedding algorithm as implemented in the BrainSpace Python toolbox (Vos de Wael et al. 2020). Diffusion embedding is a type of nonlinear dimensionality reduction based on graph theory that describes the high-dimensional connectivity data in terms of distances in a low-dimensional Euclidian space, where the distance between nodes (i.e. voxels) reflects the strength of their connections (i.e. similarity in FC patterns; for a detailed description, see Coifman and Lafon 2006). The diffusion embedding algorithm forces voxels with many and/or strong connections closer together and voxels with few and/or weak connections further apart in the embedding space (resulting in gradient maps). We extracted 10 gradients from each modality-specific matrix, but we further interrogate only the first 2 gradients as they explained considerably more variation in the data compared to the remaining gradients (see Fig. S1).

We quantified the degree of gradation in FC changes across the LIFG (including whether there are, instead, discrete changes and hard boundaries) by estimating the normalized algebraic connectivity of the similarity matrices. This value corresponds to the second largest eigenvalue of the Laplacian of the matrix and represents a descriptive index of how well connected a graph is (Fiedler 1973). It ranges from zero, which indicates that the graph comprises at least 2 completely disconnected subgraphs, to a value of 1, which suggests that the graph is characterized solely by graded differences. Thus, the normalized algebraic connectivity of the similarity matrices is indicative of whether the LIFG comprises at least 2 sharply delineated subregions or graded transitions between subregions with differences in connectivity/coactivation patterns (Bajada et al. 2020). We note that, while this value is influenced by the smoothing of neuroimaging data, a value much higher than 0 and close to the maximal value possible of 1 is unlikely to be caused only by artificially induced local gradation (Bajada et al. 2017, 2019, 2020; Jackson et al. 2020). We separately estimated the algebraic connectivity of the task-based coactivation matrix and the group task-free FC matrix. In addition, we assessed the gradation in task-free FC matrices at the participant level. This was done to avoid relying only on a gradation metric derived based on the group matrix, which is generated by subjecting the individual-level matrices to an additional transformation that may bias the gradation metric.

#### Functional characterization

While the gradient analysis can estimate the main directions of functional changes, this step alone cannot reveal the qualitative differences in the FC patterns that drive the functional organization of the LIFG. Therefore, to identify the FC differences underlying the gradient dimensions, we first defined “gradient extremes” clusters by extracting the voxels with the 20% lowest and highest gradient values (2 clusters per gradient). We selected these voxels because, in a graded map, voxels located at the gradient poles should differ most in terms of their FC patterns. In other words, probing the FC of these “gradient extremes” clusters can reveal the different connectivity patterns between which the gradients vary. Therefore, we used the “gradient extremes” clusters as seeds in whole-brain FC analyses to compare the task-free FC and task-constrained coactivation patterns that have driven the separation between the gradient extremes in the embedding space. The “gradient extremes” clusters are depicted in Fig. 1 (Step 4), and their MNI coordinates are reported in Table S1. To be clear, these clusters should not be interpreted as a hard parcellation of the LIFG but rather as a tool for gleaning a functional interpretation of the observed gradients. Indeed, similar approaches have been used in previous parcellation studies (Bajada et al. 2017; Jackson et al. 2018, 2020).

We performed seed-based resting-state FC analyses using the Python package Nilearn (Abraham et al. 2014). For each participant, we used the average resting-state fMRI time-series (concatenated across runs; Cho et al., 2021) of all voxels within each cluster as a regressor in a general linear model predicting the time-series of all gray matter voxels. The resulting cluster FC maps were z-transformed and tested for consistency across participants using a one-sample *t*-test. In addition, to identify the FC specific to each cluster, which is driving the identification of the gradient, paired-samples *t*-tests were used to generate contrast maps showing the brain regions with greater FC to one “gradient extreme” cluster than the cluster extracted from the opposite end of the same gradient (anterior vs. posterior cluster, dorsal vs. ventral cluster). The group-level FC maps were thresholded using a family-wise error (FWE)-corrected voxel-height threshold of *p* < 0.05 and the probabilistic threshold-free cluster enhancement approach as implemented in the R package pTFCE (Spisák et al. 2019). We wanted to identify the brain regions that (i) displayed greater functional coupling with one LIFG cluster than the cluster at the opposite end of the same gradient and, at the same time, (ii) were significantly coupled with the respective LIFG cluster. Therefore, the contrast maps (determined using the paired-samples *t*-tests) were masked by the significant connectivity of each cluster (determined using the one-sample *t*-tests).

To identify the coactivation patterns of each LIFG cluster, we conducted MACM analyses on seeds defined based on each task-based gradient map using the Python package NiMARE (Salo et al. 2022). Specifically, we ran ALE analyses on all studies from the NeuroQuery database that reported at least 1 activation peak within the seed (see Table S2 for the number of studies identified for each cluster) to identify the brain regions consistently involved in the studies that activate the seed. Specifically, the resulting MACM maps quantify the convergence of activation across all studies that reported activation within the seed. These maps were thresholded using an FWE-corrected voxel-level threshold of *p*< 0.05. Then, we conducted contrast analyses to identify the brain regions that coactivate more consistently with one “gradient extreme” cluster than the cluster extracted from the opposite end of the same gradient (anterior vs. posterior cluster, dorsal vs. ventral cluster). The contrast maps were thresholded using an uncorrected *p* < 0.05 threshold. To understand which brain regions display (i) greater coactivation with the cluster located at one extreme of the gradient than the other extreme and (ii) significant coactivation with the cluster, we masked the contrast maps by the significant cluster-specific MACM map (determined using independent ALE analysis).

It is important to note that we conducted contrast analyses using the same FC modality (i.e. MACM of clusters extracted from the task-based gradients, seed-based resting-state FC analyses of clusters extracted from the task-free gradients) in order to visualize the differences that have driven the gradients and not to test whether there were significant FC differences between the clusters. The noninferential and descriptive nature of these follow-up analyses circumvents analytic circularity (Eickhoff et al. 2015). Nonetheless, we repeated these sets of analyses using an independent FC modality (i.e. MACM of clusters extracted from the task-free gradients, seed-based resting-state FC analyses of clusters extracted from the task-based gradients) to confirm whether the FC maps are consistent regardless of the approach adopted to define the clusters. These analyses revealed similar FC patterns and are only reported in Figs. S9 and S10.

In line with recent recommendations (Uddin et al. 2022), we determined the network affiliations of our novel findings by comparing them with a commonly used parcellation scheme. We used the 7-network parcellation proposed by Yeo et al. (2011) as the reference atlas. For each task-free FC and task-based coactivation map, we computed the percentage of voxels that overlap with each of the 7 reference networks. The LIFG has been consistently implicated in semantic processing (Jackson 2021), which is thought to be supported by a functional network that is dissociable from other canonical networks such as the core default network (DN; Humphreys et al. 2015; Jackson et al. 2016, 2019; Branzi et al. 2020; Jung and Lambon Ralph 2023). Therefore, we also computed the overlap between our results and a mask of the semantic network (SN) obtained by Jackson et al. (2016). The reference SN map represents the set of regions that were significantly functionally coupled with the left ventrolateral anterior temporal lobe (ATL), which has been attributed a crucial role in semantic cognition (Binney et al. 2010; Lambon Ralph et al. 2017). It is of note that we are not able to dissociate between the DN and SN in these analyses (as has been done by, for example, Humphreys et al. 2015; Jackson et al. 2019) because there is a considerable degree of spatial overlap between the DN mask from Yeo et al. (2011) and the SN obtained by Jackson et al. (2016).

Lastly, to identify functional terms associated with each cluster as an index of its potential function, we conducted functional decoding analyses using the BrainMap chi-square approach as implemented in NiMARE (Salo et al. 2022). For each term in the NeuroQuery database, the consistency analysis (also known as forward inference) computes the likelihood of activation reported within the seed given presence of the term in the article’s text, whereas the specificity analysis (also known as reverse inference) estimates the posterior probability of an article containing the term given activation reported inside the seed. The results of these analyses were thresholded at *p*< 0.05 using the Benjamini–Hochberg false discovery rate correction. To aid the interpretability of the results, we retained only the terms with at least 80% likelihood of being related to cognitive functions based on raters’ annotations (Peraza et al. 2023).

## Results

### Gradient maps

**Fig. 2:**
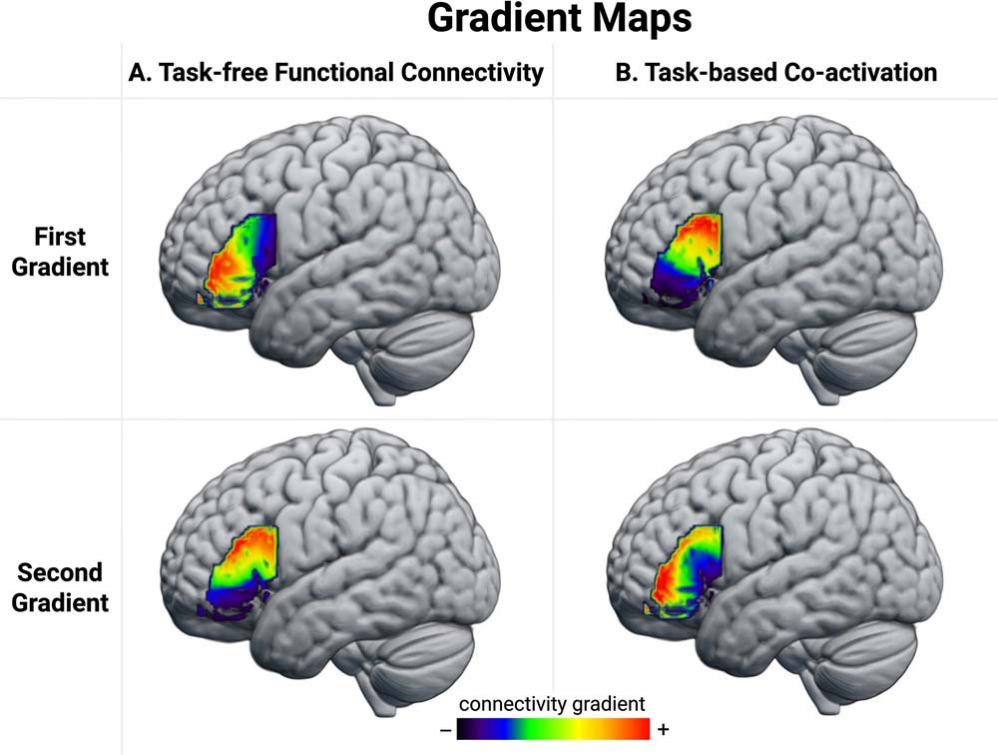
The first 2 gradient maps extracted from the A) task-free FC similarity matrix and B) task-based coactivation similarity matrix. Compared to regions represented with colors further apart on the color spectrum, regions represented using colors that are closer together show greater similarity in their A) correlation with each other over time during resting fMRI scans and B) their patterns of coactivation across tasks spanning a range of cognitive domains. The ± indicate different poles of these gradient dimensions, but the assignment to a specific end of a dimension is arbitrary. The gradient maps can be accessed and visualized at: https://identifiers.org/neurovault.collection:13053.

The first 2 task-free FC gradients were selected for further analysis because together they accounted for >50% of variance, while the lower-order gradients explained <11% of variance each (Fig. S1). The voxels’ gradient values, which reflect the similarity between their resting-state fMRI time-series, were visually coded and projected on the brain using a color spectrum from red to dark blue to reveal the pattern of change in task-free FC across the LIFG. As can be seen in Fig. 2A, the FC patterns of the LIFG are principally organized along an anterior–posterior axis that accounted for 30% of the variance. This gradient progressed from the anterior portion of the LIFG, bordering the inferior part of the inferior frontal sulcus (IFS), to the posterior region, bordering the precentral gyrus. The second gradient, which explained 25% of the variance, revealed changes in connectivity along the superior–inferior dimension. This gradient progressed from the superior part of the IFS and the precentral sulcus to the inferior portion of the IFG, bordering the lateral orbital sulcus.

The algebraic connectivity of the group similarity matrix was 0.71, suggesting a high level of gradation in task-free FC changes across the LIFG. The group similarity matrix, reordered based on the voxels’ positions along the first and second gradients, is provided in Fig. S2 and illustrates the graded change in FC across voxels in the LIFG. The graded nature of transitions was confirmed by the distribution of the algebraic connectivity of individual-level similarity matrices (Fig. S4) that had a mean of 0.89 (SD = 0.02). Moreover, inspection of distributions of gradient values within single-subject data consistently reveals an absence of large discontinuities that, once again, implies there are graded transitions rather than discrete boundaries (Fig. S6). For further discussion regarding gradients extracted at the individual level, see Section S1. A key observation, however, is that gradation seen at the group level cannot be explained by an “averaging effect” and variability in the location or extent of FC changes at the individual level. Instead, the graded nature of transitions is consistently observed at the group level and the individual subject level.

The first 2 task-based coactivation gradients were selected for further analysis because, together, they accounted for > 60% of the variance, while the lower-order gradients individually explained < 11% of the variance (Fig. S1). The principal gradient accounted for 42% of the variance and progressed along a dorsal–ventral axis from the inferior frontal junction (IFJ) to the antero-ventral region bordering the lateral orbital sulcus and inferior portion of the IFS. The second gradient explained 21% of the variance and revealed changes in connectivity that followed the rostral–caudal axis in a radial pattern progressing from the inferior portion of the pars opercularis toward the IFS. The algebraic connectivity of the coactivation similarity matrix was 0.77, suggesting that the LIFG is characterized by gradual changes in consistent patterns of coactivation across cognitive domains (see Fig. S2 for the reordered matrices). Because the unit of the task-based analysis is the study rather than the participant, the gradation cannot be assessed at the participant level as in the case of the task-free analysis reported above.

The gradients extracted from the 2 independent datasets converge on 2 principal organizational axes of the LIFG: anterior–posterior and dorsal–ventral. Visual inspection of the gradient maps suggests that the first task-free gradient and the second task-based gradient capture a similar anterior–posterior axis of functional variation, which is supported by a strong positive correlation of 0.77 between voxels’ position ranks on the 2 gradients (see Fig. S3 for the scatterplot). Likewise, the second task-free gradient and the first task-state gradient capture a similar dorsal–ventral organizational dimension. This observation is supported by a strong positive correlation of 0.7 between voxels’ position ranks on the 2 gradients (Fig. S3). The orders in which these gradients appear are switched between the task-free and task-constrained FC data, and this is because of a difference in the relative amount of variance explained by each gradient. Because it is subtle relative to the similarities, this difference could be attributable to noise but it may also reflect meaningful differences in the connectivity revealed by task-free and task-constrained mental states (Eickhoff and Grefkes 2011).

### Functional characterization

#### Differential task-free FC patterns

**Fig. 3:**
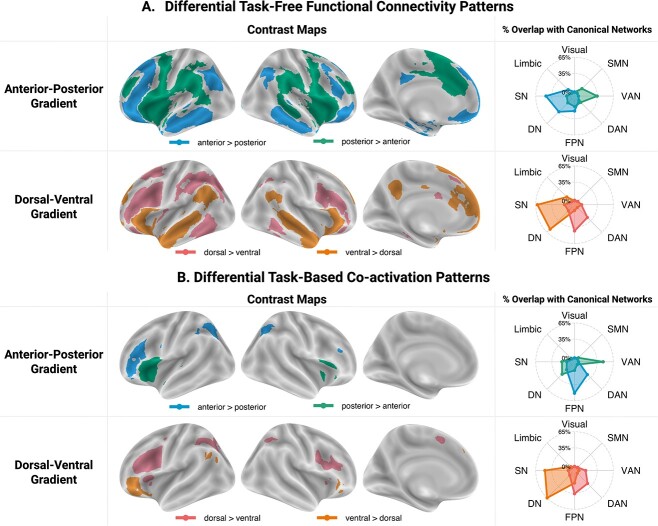
Results of contrast analyses between A) the task-free connectivity patterns (derived using seed-based resting-state FC analyses) of the IFG clusters located at the extremes of the anterior–posterior and dorsal–ventral task-free gradients and B) task-constrained coactivation patterns (derived using MACM analyses) of the IFG clusters located at the extremes of the anterior–posterior and dorsal–ventral task-based gradients. These contrast maps were masked using cluster-independent maps. The spider plots in the right column show the percentage of voxels in each contrast map that overlap with each of the 7 canonical networks from Thomas Yeo et al. (2011), as well as the SN from Jackson et al. (2016; available at: github.com/JacksonBecky/templates). Note that percentage values are relative to the size of each contrast map; therefore, only the relative patterns of overlaps within each contrast map are of interest and direct comparisons between the network affiliations of different contrast maps are misleading. The contrast maps can be accessed at: https://identifiers.org/neurovault.collection:13053.

We contrasted the whole-brain resting-state connectivity patterns of the clusters located at the extremes of the anterior–posterior task-free gradient. This revealed differences in their functional coupling with a bilateral and distributed set of brain regions (Fig. 3A; Table S3). The anterior cluster showed stronger FC with frontal regions, including the right IFG (pars orbitalis), bilateral IFS, dorsal and orbital portion of the middle frontal gyrus (MFG), superior frontal gyrus (SFG), medial prefrontal cortex (mPFC), and orbitofrontal cortex (OFC), with parietal regions in the posterior cingulate cortex (PCC), angular gyrus (AG), and inferior parietal lobule (IPL), with temporal regions along the length of the middle and inferior temporal gyri (MTG/ITG), in the fusiform gyrus (FG), and left hippocampus. In contrast, the posterior cluster showed stronger FC with frontal regions in the right IFG (pars opercularis), the middle portion of the MFG, precentral gyrus, pre-supplementary motor area (pre-SMA), anterior and middle cingulate cortex (ACC), and insula, with posterior cortical regions in the supramarginal gyrus (SMG) and posterior superior temporal sulcus, and with basal ganglia.

Comparison between the task-free FC of the dorsal and ventral clusters revealed stronger coupling between the dorsal LIFG and frontal regions in bilateral IFS and IFJ, left MFG, and pre-SMA, parietal cortex in bilateral IPS and left IPL, and temporal cortex in bilateral posterior ITG and left FG (Fig. 3A; Table S4). In contrast, the ventral LIFG showed increased connectivity to the frontal cortex in the right IFG (pars triangularis and pars orbitalis), bilateral SFG, mPFC, and ACC, to the precuneus, a swathe of temporal cortex progressing from the bilateral ventrolateral ATL through the MTG toward the AG, and to the left hippocampus.

Comparison between the cluster-specific task-free FC patterns and canonical networks indicate stronger functional coupling between the anterior LIFG and regions falling within the bounds of the SN/DN and the frontoparietal network (FPN), and between the posterior LIFG and brain regions associated with the ventral attention network (VAN) and somatomotor network (SMN). The dorsal LIFG showed stronger FC with regions of the FPN and dorsal attention network (DAN), whereas the ventral LIFG showed a preference for SN/DN regions. Additional conjunction analyses showed that both the anterior and posterior clusters are coupled with regions of the FPN and SN/DN and that both the dorsal and ventral clusters are functionally connected mainly with SN/DN regions (Fig. S8a; Tables S3 and S4).

#### Differential task-constrained coactivation patterns

The anterior LIFG showed increased consistent coactivation across a wide variety of tasks with frontal regions in the right IFS and precentral gyrus, and with bilateral IPS and left posterior ITG, whereas the posterior LIFG coactivated more with the right IFG, bilateral anterior insula, and left superior temporal gyrus (Fig. 3B; Table S5). The dorsal cluster coactivated more with frontal cortex in the right IFJ, bilateral precentral gyrus, dorsal anterior insula, and pre-SMA, and with the IPS, and left posterior FG (Fig. 3B; Table S6). In comparison, the ventral cluster showed increased coactivation with the right IFG (pars orbitalis), and left mPFC, MTG, and AG. Given the conservative threshold applied to the independent maps, we also looked at the whole contrast maps without masking by these independent maps. These additionally revealed more consistent coactivation of the posterior cluster with the bilateral STG and of the ventral cluster with the bilateral ATL, precuneus, and left AG (Fig. S7).

Comparison between the cluster-specific task-based coactivation patterns and canonical networks shows that the anterior LIFG cluster coactivates more consistently with brain regions that are part of the FPN and DAN, whereas the posterior LIFG cluster coactivates mainly with regions associated with the VAN and, when additional masking is not applied, the SMN. The dorsal cluster coactivates preferentially with regions of the FPN and DAN, whereas the ventral LIFG cluster shows stronger coactivation with the DN/SN. Additional conjunction analyses showed overlap between the coactivation maps of the anterior and posterior clusters and those of dorsal and ventral clusters primarily in regions of the FPN (Fig. S8b; Tables S5 and S6).

The FC analyses performed on clusters extracted from the gradient maps derived using the independent dataset (i.e. seed-based FC analyses of clusters derived using NeuroQuery studies and MACM analyses of clusters derived using task-free fMRI time-series), which were conducted to assess the robustness of the results across different strategies for defining seeds, revealed a similar pattern of results (Figs. S9 and S10).

#### Comparison between the task-free and task-based FC patterns

In sum, the task-free and task-based analyses implicate overlapping regions, although the clusters identified in the task-based analyses were less extensive. Specifically, the anterior LIFG was connected with executive control regions (e.g. IFJ, IPS; Fedorenko et al. 2013; Camilleri et al. 2018; Assem et al. 2020b), but, in the task-free maps, it was also connected to regions implicated in semantic cognition (e.g. ATL, AG; Binder and Desai 2011; Lambon Ralph et al. 2017). Further, the posterior LIFG was connected to areas that have been ascribed important roles in sensorimotor processing, as well as in phonological and articulatory linguistic processes (e.g. bilateral STS/STG, but in the task-free maps, it was also connected to motor and premotor cortices, SMA, MFG, and SMG; Vigneau et al. 2006; Hickok and Poeppel 2007; Hickok 2009; Hartwigsen et al. 2010; Ueno et al. 2011; Price 2012). This cluster was also connected to regions considered crucial for salience processing (e.g. anterior insula, but in the task-free results also to dorsal ACC; Menon and Uddin 2010; Uddin 2015). The dorsal LIFG was connected to regions that are implicated in executive function (e.g. IFJ, MFG, IPS; Fedorenko et al. 2013; Camilleri et al. 2018; Assem et al. 2020b), whereas the ventral LIFG was connected with a set of regions ascribed key roles in semantic and episodic memory (e.g. ATL, medial temporal lobe, AG; (Irish and Piguet 2013; Lambon Ralph et al. 2017).

Despite the similarities in the regions implicated, there were some differences in the network affiliations derived from the task-free and task-based analyses. However, comparing the network affiliations of the different contrast maps directly is not possible because (i) the overlap index depends on the size of the maps, which differs considerably between the task-free and task-based analyses, and (ii) there are differences between the seeds upon which the task-free and task-based analyses are based (see Fig. 1; e.g. the task-based anterior seed extends across the length of the IFS and overlaps with the dorsal LIFG seed, whereas the anterior seed used for the task-free analysis does not). Therefore, we will focus the interpretation on the similarities.

The dorsal LIFG connected to FPN and DAN regions, 2 networks that contribute to the task-general multiple demand network (MDN; Majerus et al. 2018; Assem et al. 2020b). In contrast, the ventral LIFG was affiliated mainly with the DN/SN. The DN and SN cannot be distinguished in our assessment given the high degree of spatial overlap between the masks used. However, we note that both the task-free and the unmasked task-based results suggest strong coupling with the ATL, a key hub of semantic knowledge (Binney et al. 2010; Lambon Ralph et al. 2010), as well as with the left hippocampus/parahippocampal gyrus, known to be important for episodic memory (Burgess et al. 2002; Dickerson and Eichenbaum 2010). As such, the dorsal–ventral organizational dimension seems to distinguish between domain-general control networks at the dorsal end and memory-related networks at the ventral end.

The posterior LIFG showed a preference for the VAN, suggestive of a role in perceptually driven cognition (Corbetta and Shulman 2002; Corbetta et al. 2008). The anterior LIFG showed a preference for regions that overlap with the FPN, consistent with a role in cognitive control (Assem et al. 2020a). The task-free data revealed additional strong coupling with regions that are part of the DN/SN. The task-based analyses might have led to less extensive association with the DN because this network is known for its tendency to deactivate in response to various task demands (Shulman et al. 1997; Mazoyer et al. 2001; Buckner et al. 2005), but it tends to activate during mind-wandering states that frequently occur during resting-state scans (Smallwood et al. 2021). Nonetheless, there is evidence that the DN works with the FPN in support of some types of goal-directed cognition (Spreng et al. 2010, 2013, 2014; Wang et al. 2021) and that it contributes to cognitive control (Crittenden et al. 2015). As such, the anterior–posterior organizational dimension seems to distinguish between higher-order transmodal networks at the anterior edge and perceptually driven networks at the posterior edge.

#### Functional decoding

The functional decoding results suggest possible functional associations of the different LIFG clusters. We identified a set of common terms that suggest functional associations shared by all clusters. All 4 LIFG clusters were significantly associated with terms related to semantic and linguistic processing, including *lexical*, *linguistic*, *language, language comprehension, sentence comprehension, reading, orthographic, phonological, lexical decision,* and *retrieval semantic information/memory/knowledge*. In addition, we identified associations with distinct terms that hint at the functional dissociations between the gradient edges. Compared to the posterior cluster, the anterior cluster was additionally associated with the terms *executive (function)* and *memory retrieval,* in line with a role in higher-order cognitive control processes *.* In contrast, the posterior cluster was associated with terms related to lower-order perceptual and motor processing, such as *movement, recognition,* and *auditory,* as well as speech-related terms, such as *phonetic* and *vocal.* Compared to the ventral cluster, the dorsal cluster was associated with terms related to a wide range of cognitive/behavioral domains and input modalities, including *visual, auditory, visuospatial, working memory, executive, social, reward,* and *mood.* In contrast, the ventral cluster was associated with terms such as *memories, mentalizing, reappraisal,* and *autobiographical,* which are suggestive of the purported internally oriented functions of the DN (Andrews-Hanna et al. 2014; Smallwood et al. 2021). While functional decoding approaches can provide pointers to the potential functional associations of these regions, it is important to note that the specificity of the results is limited by the drawbacks of automated data mining tools like NeuroQuery. These include the aggregation of all contrasts reported in an article, regardless of the cognitive aspects they isolate (Dockès et al. 2020). Therefore, interpretation should focus on the overall patterns that emerge in the decoding analysis, rather than the associations of individual terms. Detailed lists of the functional associations are presented separately for forward and reverse inference analyses and task-free and task-based clusters in Figs. S11 and S12.

**Fig. 4:**
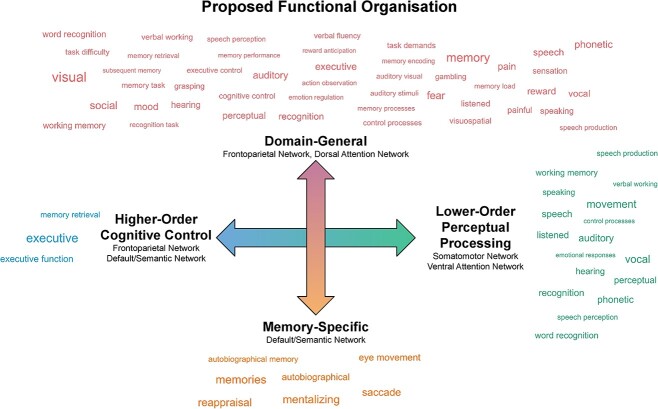
Schematic representation of the proposed functional organization of the LIFG. The word clouds illustrate functional terms associated with parts of the LIFG located at the extremes of the gradients (derived from task-free and task-based FC) on the basis of forward or reverse inference functional decoding analyses. Terms associated with the anterior but not posterior aspect are shown in blue, those associated with the posterior but not anterior aspect in green, the dorsal but not ventral aspect in red, and with the ventral but not dorsal aspect in orange. The size of the word reflects the effect size of the association. The anterior–posterior organizational axis is represented by the horizontal blue-to-green arrow, and we interpret the sets of associations to reflect a shift from lower-order perceptual processing (posterior LIFG) via affiliation with the SMN and VAN, to higher-order cognitive control (anterior LIFG) via affiliation with the FPN and DN. The dorsal–ventral axis is represented by the vertical orange-to-red arrow, and we interpret the sets of associations to reflect a shift from domain-general executive functions (dorsal LIFG) via affiliation with the FPN and DAN to cognitive control of information stored in long-term memory (ventral LIFG) via affiliation with the DN/SN.

Figure 4 summarizes the functional decoding results that were consistent for the clusters extracted from the task-free and task-based gradients (e.g. terms associated with both the anterior edge of the task-free gradient and the anterior extreme of the task-based gradient). It also includes a schematic of the proposed functional organization, which takes into account the results of the FC contrast analyses, the network affiliations, and the functional decoding, as well as previous literature reviewed in detail in the Discussion.

## Discussion

The present study made the first attempt to use data-driven gradient analyses of FC data to elucidate the functional organization of the LIFG. We specifically aimed to (i) map the principal axes of change in function and (ii) determine whether these shifts are graded or discrete. In the following 2 sections, we shall summarize our novel findings and then discuss their functional significance.

### Graded topographical organization of the LIFG along 2 principal axes

Our analyses converged upon 2 key findings. First, the FC across the LIFG is principally organized along 2 orthogonal axes. One of these axes is oriented in an anterior to posterior direction and driven by stronger coupling with the FPN and DN in the rostral aspect and with the VAN and SMN at the caudal end. The second arose along a ventral to dorsal orientation and reflected greater connectivity of ventral LIFG to the DN, whereas dorsal regions abutting the IFS/IFJ were more tightly coupled with the FPN and DAN. These differential patterns of FC are in line with previous investigations (Kelly et al. 2010; Clos et al. 2013; Neubert et al. 2014; Barredo et al. 2016; Davey et al. 2016; Jakobsen et al. 2016, 2018; Muhle-Karbe et al. 2016; Wang et al. 2020) and suggest that the LIFG interfaces between distinct large-scale functional networks, consistent with its proposed role as a cortical hub (Buckner et al. 2009; Sepulcre et al. 2012).

Our second key finding is that FC of the LIFG shifts in a graded manner. Put another way, the algebraic connectivity of the similarity matrices revealed that FC is not consistent with abrupt boundaries and discrete functional parcels in the LIFG. Our findings are the first direct demonstration of this, but they are consistent with both contemporary descriptions of LIFG connectivity based on intraoperative cortico-cortical evoked potentials (Nakae et al. 2020) and structural properties (Thiebaut de Schotten et al. 2017), as well as classical descriptions that include a fan-shaped set of anatomical projections emanating from the IFG into the lateral temporal lobe (Dejerine and Dejerine-Klumpke 1895).

Overall, our findings are compatible with previous parcellations despite key differences in the methodological approaches (Klein et al. 2007; Kelly et al. 2010; Clos et al. 2013; Neubert et al. 2014; Wang et al. 2020; Blazquez Freches et al. 2021; Abdallah et al. 2022). This includes those that have taken a “hard” clustering approach to parcellating the left prefrontal cortex (PFC). For example, Neubert et al. (2014) parcellated the PFC based on structural connectivity and found that the LIFG fractionated into discrete subdivisions positioned along the anterior–posterior dimension. The connectivity of these parcels was distinct from those situated dorsally in the adjacent IFS and IFJ, which implies a further dorsal–ventral dimension of organization. The coexistence of these 2 axes of LIFG organization is also apparent in hard parcellations of the LIFG (Clos et al. 2013; Wang et al. 2020), its right hemisphere homolog (Hartwigsen et al. 2019), and more encompassing parcellations of cortex (Glasser et al. 2016). Of course, the results of graded and hard parcellation are not identical because hard parcellations (i) force voxels that are part of intermediate regions with gradually changing connectivity to be within the borders of discrete clusters (Haueis 2012; Bajada et al. 2017) and (ii) require the a priori specification of the number of clusters that are to be identified, perhaps making them insensitive to finer details. However, the 2 superimposed yet orthogonal modes of organization identified here (cf. Blazquez Freches et al. 2021; Abdallah et al. 2022) could have driven the results of prior hard parcellations of the LIFG.

### The putative functional significance of the LIFG’s functional connectivity gradients

Taken together, the cluster-specific FC patterns and functional decoding results paint a coherent picture regarding the functional significance of the graded connectivity patterns that appear across the LIFG. On this basis, and in conjunction with the results of previous functional neuroimaging studies, we propose the following interpretation, which is illustrated schematically in Fig. 4. First, the dorsal–ventral axis might reflect a functional transition from domain-general executive function (dorsal LIFG) to domain-specific control of meaning-related representations (ventral LIFG). Second, the anterior–posterior axis might reflect a shift from perceptually driven processes (posterior LIFG) to higher-level transmodal control (anterior LIFG). We discuss this proposal in further detail below.

The dorsal LIFG was functionally coupled with regions that comprise the FPN and DAN. These 2 networks contribute to a wide variety of task demands that span multiple cognitive domains (Cole et al. 2013; Assem et al. 2020b). In contrast, the ventral LIFG was preferentially affiliated with the DN, including brain regions that have been ascribed key roles in episodic memory, like the medial temporal lobes (Dickerson and Eichenbaum 2010; Eichenbaum et al. 2012; Ekstrom and Ranganath 2018; Sugar and Moser 2019), and semantic cognition, like the ATLs (Binney et al. 2010; Binder and Desai 2011; Lambon Ralph et al. 2017). The ATLs also make important contributions to episodic/autobiographical memory (Svoboda et al. 2006; Irish et al. 2012; Irish and Vatansever 2020), perhaps providing the semantic scaffold necessary for shaping and constraining the encoding and recollection of past experiences (Irish and Piguet 2013; Renoult et al. 2019). Thus, the shift in FC toward ventral IFG subregions might reflect a specialization toward the application of cognitive control to prior knowledge. Indeed, it has been proposed that the LIFG, as a whole, sits in a unique position at the intersection of the MDN and the DN and that this makes it ideally suited for implementing demanding operations on meaning-related representations from memory (Davey et al. 2016; Chiou et al. 2023). Consistent with this, the LIFG responds reliably to an increased need for the controlled access to stored semantic information across a wide range of experimental paradigms (Diveica et al. 2021; Jackson 2021), extending to those requiring the retrieval of episodic memories (Vatansever et al. 2021). However, it is increasingly apparent that there are finer-grained functional distinctions within the LIFG; dorsal LIFG regions near IFS/IFJ overlap with the MDN and are engaged by control demands that are common across many cognitive tasks/domains (Fedorenko et al. 2013; Assem et al. 2020b; Hodgson et al. 2021), which may include phonology (Poldrack et al. 1999; Hodgson et al. 2021), whereas the ventral LIFG contributes selectively to challenging semantic tasks (Whitney et al. 2011, 2012; Gao et al. 2021). One possible explanation is that ventral LIFG is specifically involved in controlled semantic retrieval processes as opposed to domain-general selection mechanisms, which are under the purview of dorsal LIFG regions (Badre and Wagner 2007; Barredo et al. 2015; but see Thompson-Schill et al. 1997; Crescentini et al. 2010; Snyder et al. 2011). Alternatively, the cognitive mechanisms implemented might be equivalent, but connectivity differences mean that they operate on distinct sets of inputs/outputs (Thompson-Schill et al. 2005).

The anterior and posterior LIFG clusters were each affiliated with networks that occupy different positions along a macroscale cortical hierarchy that transitions from the sensorimotor to transmodal cortex (Margulies et al. 2016). Specifically, the posterior LIFG was coupled with the VAN and SMN, which are positioned at the lower edge of the principal cortical gradient and process inputs from the external environment (Corbetta et al. 2008; Menon and Uddin 2010). In contrast, anterior LIFG was preferentially coupled with regions of the FPN and DN, which are positioned toward the top end of the cortical hierarchy (Margulies et al. 2016). The anatomical and functional separation of anterior LIFG regions from sensorimotor systems might be requisite for the implementation of perceptually decoupled, temporally extended, and higher-order cognitive control (Fuster 2001; Kiebel et al. 2008; Taylor et al. 2015; Raut et al. 2020). This interpretation is consistent with the proposal that the PFC is characterized by a posterior–anterior gradient of hierarchical control (for a review, see Badre 2008; Badre and Desrochers 2019), which was motivated by studies showing that the anterior PFC is preferentially engaged by tasks that require generalization over an extended set of rules, integration of a larger number of dimensions, and/or contexts sustained over longer periods of time (for an investigation focused on the LIFG, see Koechlin and Jubault 2006; also Koechlin et al. 2003; Badre and D’Esposito 2007; Bahlmann et al. 2015; Nee and D’Esposito 2016). In the language domain, it has been suggested that the LIFG has a key role in the integration of subordinate lexical elements into superordinate representational structures (e.g. from phonemes to words to sentences to conceptual gestalts) and that this reflects a caudal–rostral functional gradient from phonological to syntactic to conceptual processing (Hagoort 2005; Uddén and Bahlmann 2012; also see Asano et al. 2021). Our results can account for this functional distinction, as preferential coupling with perceptual-motor networks can explain the reliance of phonology on posterior LIFG, whereas increased FC with the SN explains the involvement of antero-ventral LIFG in semantic processing. The stronger FC with the posterior superior temporal cortex might make the more posterior aspect of the LIFG also suited for implementing syntactic binding computations (Zaccarella et al. 2017; Zaccarella and Friederici 2017), particularly in the case of highly automated linguistic tasks that require lower levels of cognitive control (Jeon and Friederici 2013, 2015).

### Concluding remarks

Our analyses revealed 2 main axes of organization in LIFG function, in anterior–posterior and dorsal–ventral orientations, which is consistent with broader proposals concerning the whole PFC (Petrides 2005; Abdallah et al. 2022). Moreover, our results suggest that functional differentiation across the LIFG occurs in a graded manner, and we were not able to find any clear evidence for discrete functional modules. Crucially, we replicated the principal gradients using 2 independent measures of FC, which suggests that our results are not dependent on idiosyncrasies of the datasets and instead reflect stable, generalizable properties of LIFG organization. The high degree of cross-modal similarity also suggests that a comparable LIFG functional organization underpins divergent mental states. Therefore, our work provides a spatial framework that can help us understand the contribution of the LIFG to multiple cognitive domains. Future work is needed to directly probe the functional significance of these organizational dimensions and assess the compatibility of our findings at different spatial scales (e.g. cellular) and within other neuroimaging modalities (e.g. tractography) such that it is possible to arrive at an integrated account of the functional organization of the LIFG.

## Supplementary Material

Diveicaetal_LIFGgradients_Supplementary_Information_Final_bhad373

## Open science practices and data/code availability statement

We used open data available via the Human Connectome Project (https://www.humanconnectome.org/) and the NeuroQuery database (https://neuroquery.org/). The code used for data preprocessing and analysis relies on open source software (e.g. Abraham et al. 2014; Salo et al. 2022) and can be accessed at: https://github.com/DiveicaV/LIFG_Gradients and https://osf.io/u2834/. The gradient and functional connectivity maps can also be accessed and visualized via NeuroVault at: https://identifiers.org/neurovault.collection:13053. Due to its exploratory nature, the study was not preregistered.
