# Supplementary material for: Graded functional organization in the left inferior frontal gyrus: evidence from task-free and task-based functional connectivity
Source: Cereb Cortex. 2023 Oct 13;33(23):11384–99. doi: 10.1093/cercor/bhad373 (PMC10690868; doi:10.1093/cercor/bhad373)
Supplement: Diveicaetal_LIFGgradients_Supplementary_Information_Final_bhad373 [file diveicaetal_lifggradients_supplementary_information_final_bhad373.pdf]

Supplementary Information

for

Graded functional organisation in the left inferior frontal gyrus: evidence from task-free and task-based functional connectivity

Veronica Diveica, Michael C. Riedel, Taylor Salo, Angela R. Laird, Rebecca L. Jackson &

Richard J. Binney

List of Contents

Figure S1 ………………………………………………………………………………. 2
Figure S2 ………………………………………………………………………………. 3
Figure S3 ………………………………………………………………………………. 4
Section S1 ……………………………………………………………………………... 5
Figure S4……………………………………………………………………… 6
Figure S5……………………………………………………………………… 7
Figure S6……………………………………………………………………… 8
Figure S7 ………………………………………………………………………………. 9
Figure S8 ………………………………………………………………………………. 10
Figure S9 ………………………………………………………………………………. 11
Figure S10 . ……………………………………………………………………………. 12
Figure S11 . ……………………………………………………………………………. 13
Figure S12 …………………………………………………………………………… 14
Table S1 ………………………………………………………………………………. 15
Table S2 ………………………………………………………………………………. 15
Table S3 ………………………………………………………………………………. 16
Table S4 ………………………………………………………………………………. 17
Table S5 ………………………………………………………………………………. 18
Table S6 ………………………………………………………………………………. 18
References ……………………………………………………………………………... 19

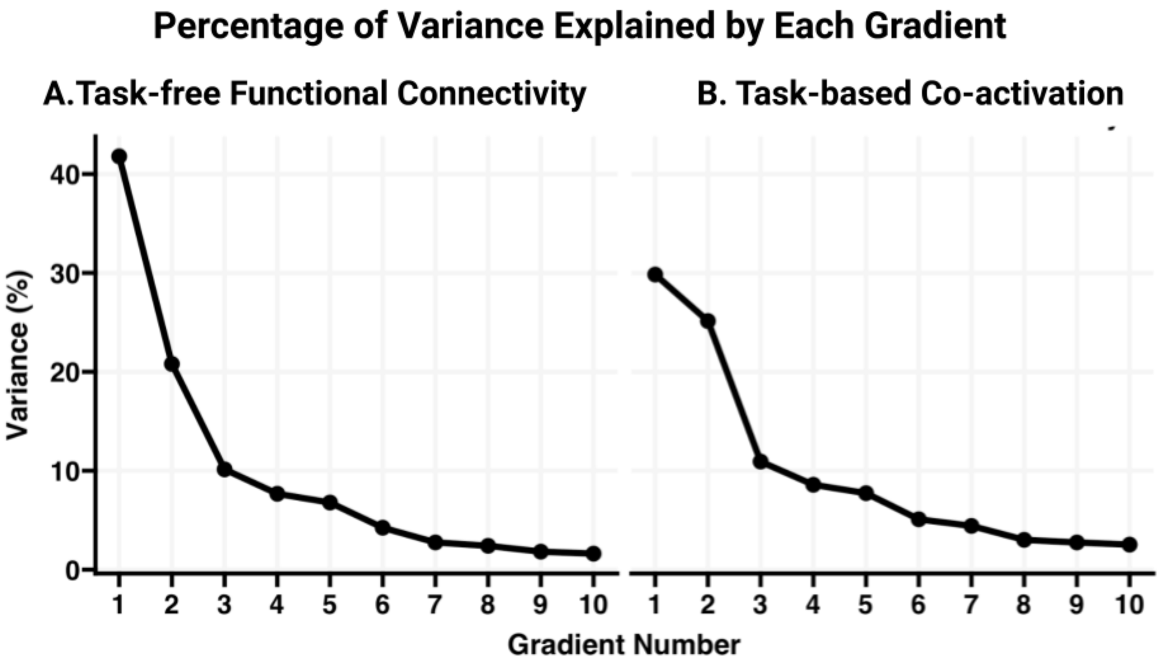

Figure S1. Percentage of variance explained by the 10 gradients derived from A) the task-free functional connectivity data and B) the task-based co-activation patterns.

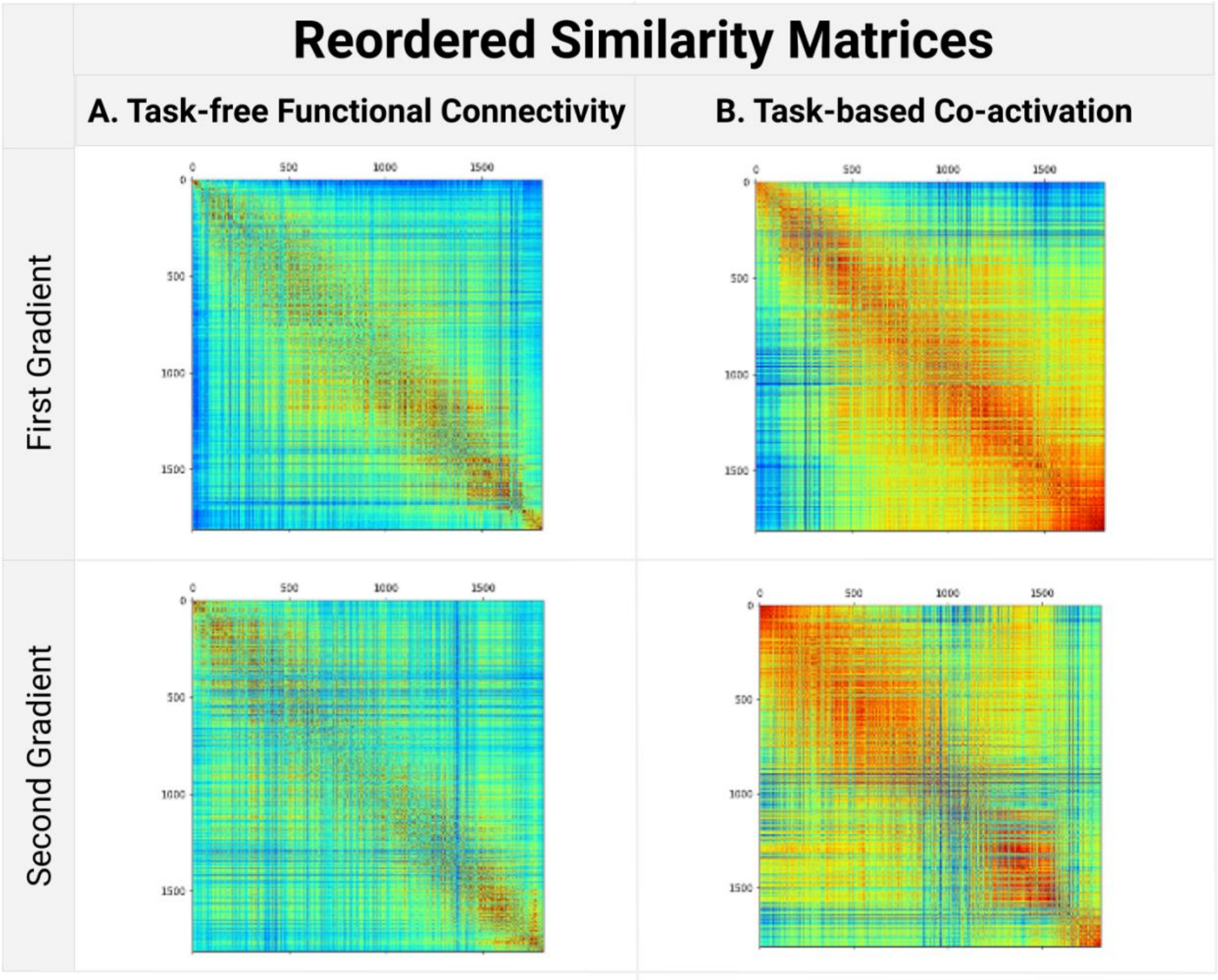

Figure S2. Similarity matrices reordered based on the voxels' positions along the first and second gradients. A) Reordered task-free FC group matrix. B) Reordered task-based co-activation matrix. Visual inspection of the reordered matrices suggests a high degree of gradation in the main axes of functional connectivity change across the left IFG.

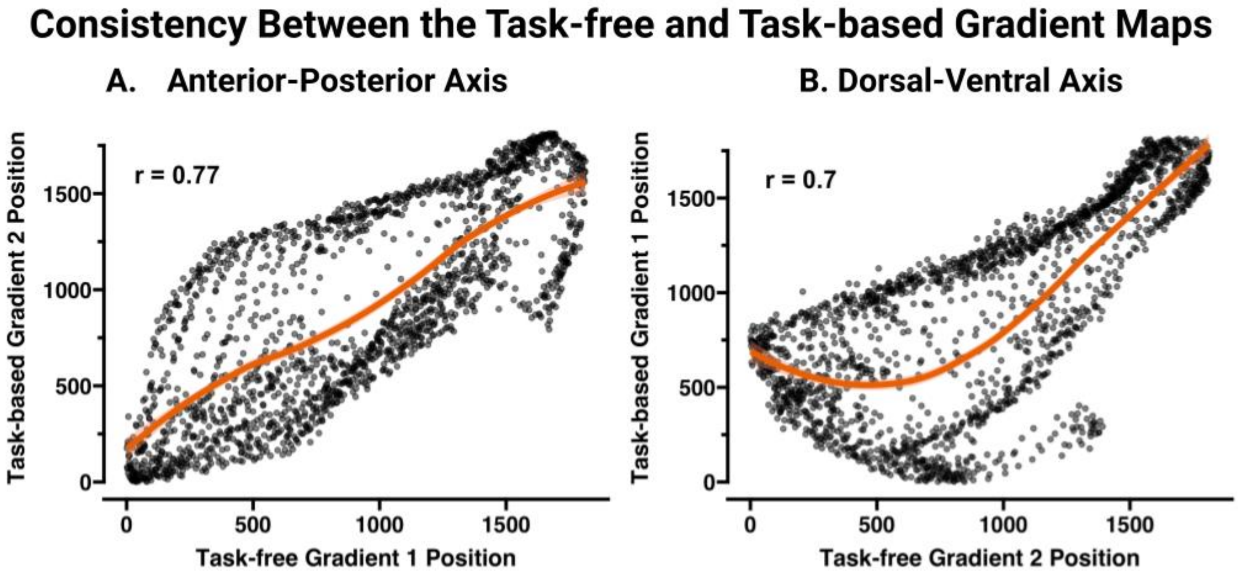

Figure S3. Scatterplots illustrate the relationship between voxels' positions in the resting-state gradients and the task-state gradients. The loess lines depicted in orange highlights the functional relationship. A. Voxels' ranks on the anterior-posterior (first) task-free gradient are plotted against their ranks on the anterior-posterior (second) task-based gradient. B. Voxels' ranks on the dorsal-ventral (second) task-free gradient are plotted against their ranks on the dorsal-ventral (first) task-based gradient. The r values represent the product-moment correlation coefficients and suggest strong relationships between the gradients extracted from the two independent FC datasets.

Section S1. Individual-level gradient analyses

The group-level analyses reveal evidence of gradual changes in functional variation across the LIFG. It is possible that, to some extent, these group-level graded patterns reflect an averaging over individual-level variation in terms of the location and extent of FC changes when, in fact, the changes are discrete (i.e., delineated by sharp boundaries). To investigate this possibility, we computed the gradation metric separately for each participant's connectivity matrix. The distribution of individual-level gradation values (see Figure S4) had a mean of 0.89 (SD = 0.02), showing that in all 150 individual brains investigated there is evidence of gradual changes in FC.

Furthermore, in Figures S5-S6, we plotted the relationship between a voxel's position on the first and second gradients. Should functional organisation of the LIFG be characterized by discrete subregions with sharp borders, then these plots would reveal abrupt discontinuities in the plotted gradient values along with visibly separable clusters of voxels. Indeed, the distance between voxels' gradient values has been used as a measure of gradation, with larger distances indexing more sudden changes in connectivity (see Cerliani et al., 2012). However, such a pattern was absent both at the group level (see Figure S5), and at the level of individual participants (Figure S6), and the distributions of gradient values are more consistent with gradual changes in FC.

Finally, in Figure S6 we confirmed that, despite some expected variability across participants (Kong et al., 2019), both principal axes of variation (along the dorsal-ventral and anterior-posterior spatial dimensions) can be identified at the level of individual brains, as well as at the group level.

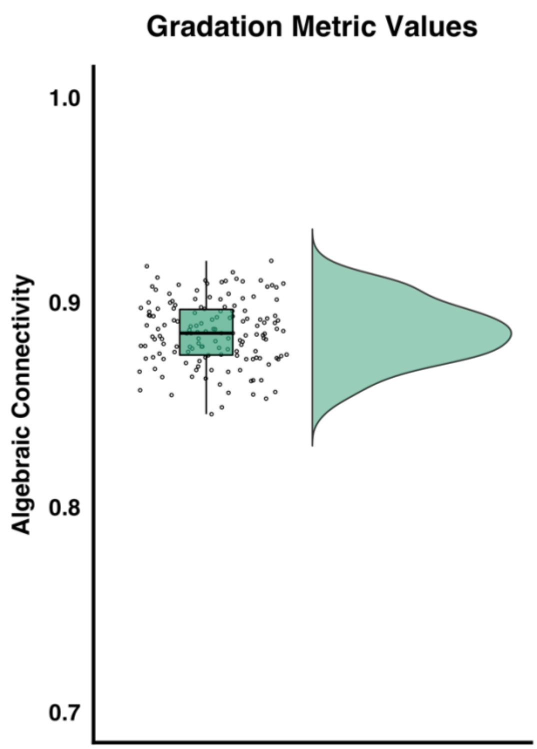

Figure S4. The distribution of the algebraic connectivity values (equivalent to the second largest eigenvalue of the Laplacian of the similarity matrix) obtained per participant in the task-free functional connectivity assessment is plotted alongside individual datapoints and a boxplot highlighting the median, 25th and 75th quartiles. Values near 0 reflect the existence of hard clusters, whereas higher numbers suggest a graded change in functional connectivity. Note that the y-axis starts at 0.7, which is above the midpoint of possible values. The gradation metric values suggest that the left IFG is characterized by graded changes in task-free functional connectivity that can be observed at the individual participant level.

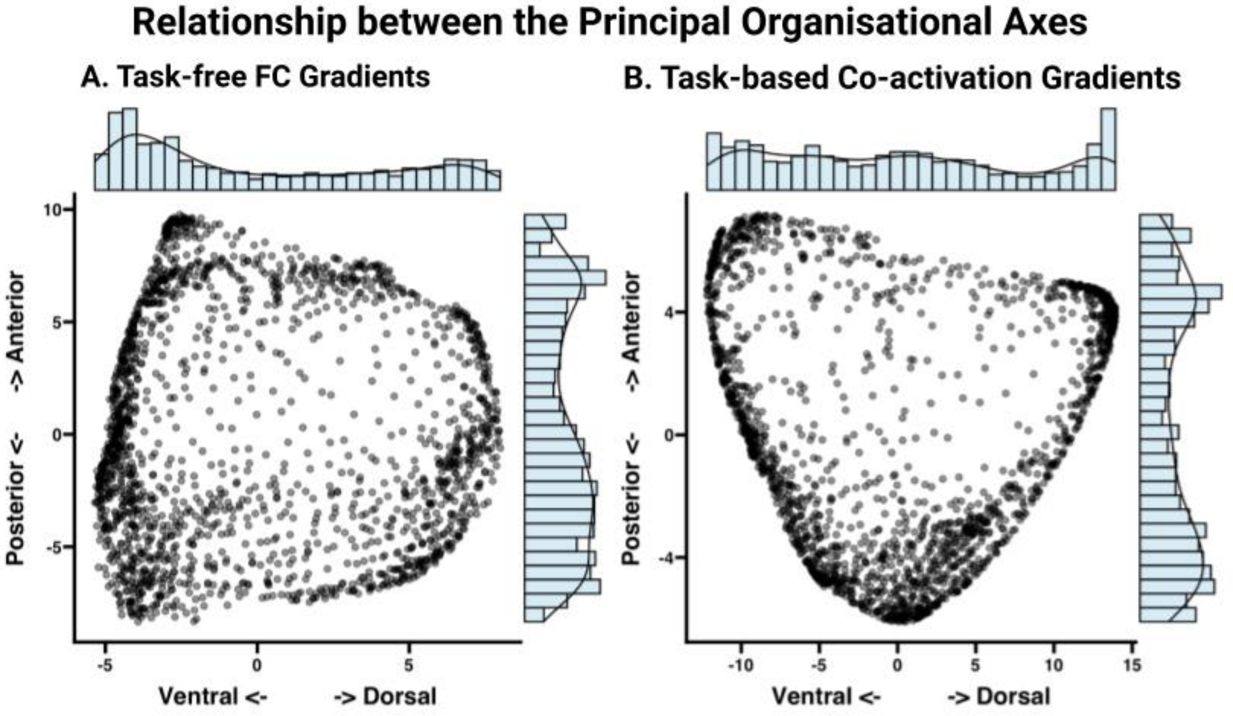

Figure S5. Scatterplots illustrate the relationship between voxels' gradient values on the first two connectivity embedding gradients extracted from A) task-free functional connectivity and B) task-based co-activation patterns. Histograms and density plots depicting the distribution of gradient values are presented on the respective axes.

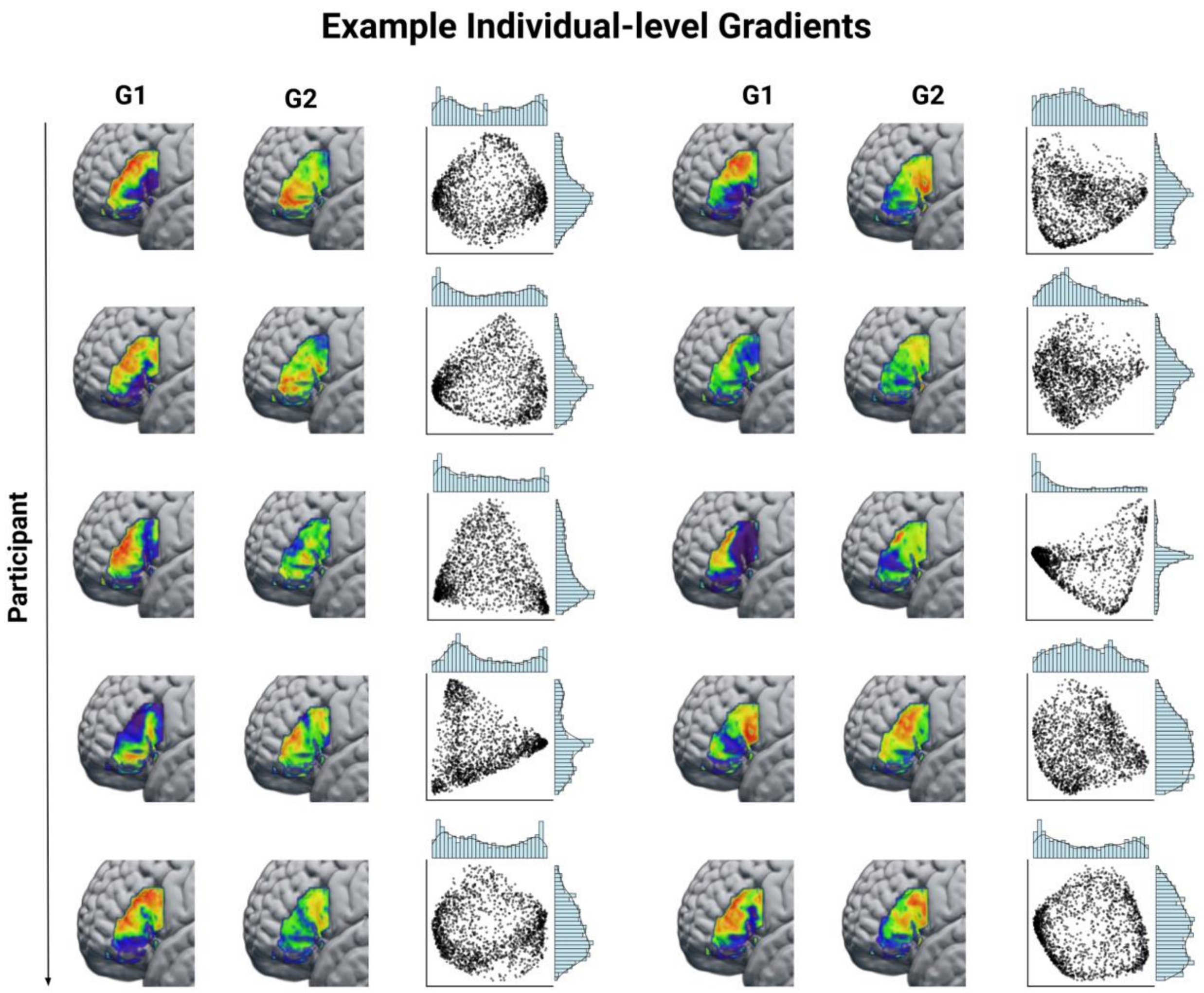

Figure S6. Illustrates the gradients extracted from 10 example participants. The G1 column shows the principal axis of variation in task-free functional connectivity, whereas the G2 column shows the secondary axis of variation. The scatterplots illustrate the relationship between voxels' gradient values on the first gradient, G1 (x axis), and the second gradient, G2 (y axis). Histograms and density plots depicting the distribution of gradient values are also presented on the respective axes. This figure demonstrates (i) that anterior-posterior and dorsal-ventral gradients can be identified at the individual participant level as well as the group level, and (ii) an absence of clearly separable clusters of voxels at the single-subject level suggesting a lack of abrupt changes in FC (i.e., there are graded changes in FC).

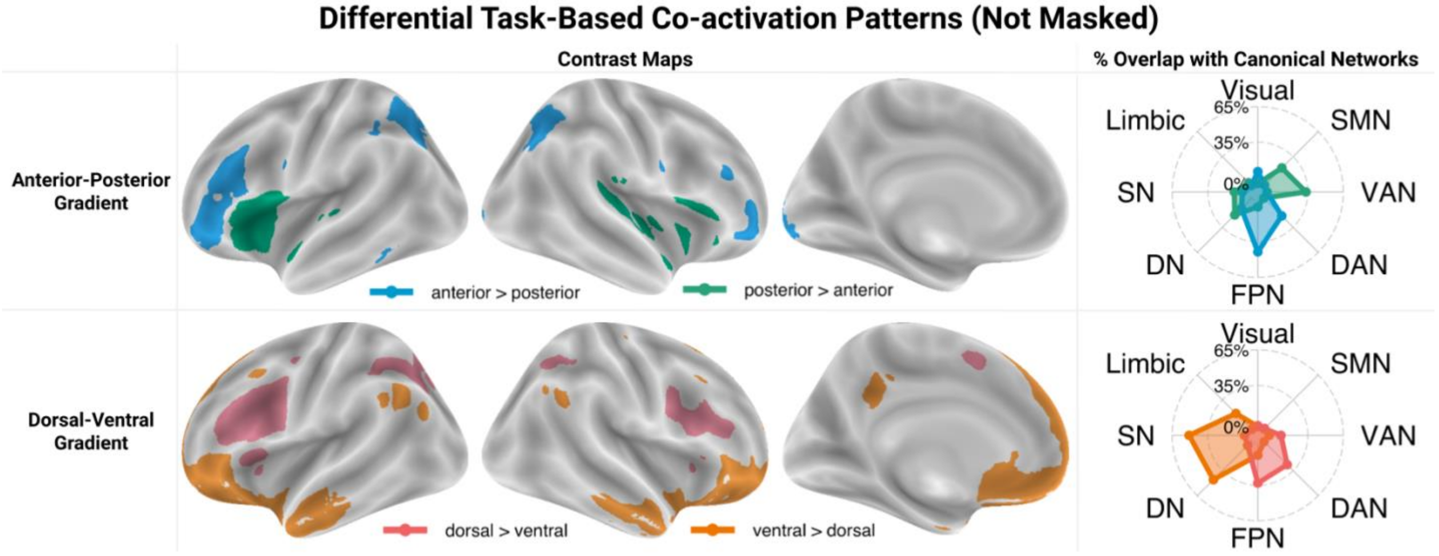

Figure S7. Results of contrast analyses between task-constrained co-activation patterns (derived using MACM analyses) of the IFG clusters located at the extremes of the anterior-posterior and dorsal-ventral task-based gradients. Unlike in the main text, these contrast maps were not masked using independent MACM maps. The spider plots in the right column show the percentage of overlap between the contrast maps and canonical networks from Yeo et al. (2011), as well as the semantic network from Jackson et al. (2016), which is comprised of regions that are functionally coupled with the ventrolateral anterior temporal lobe semantic hub at rest.

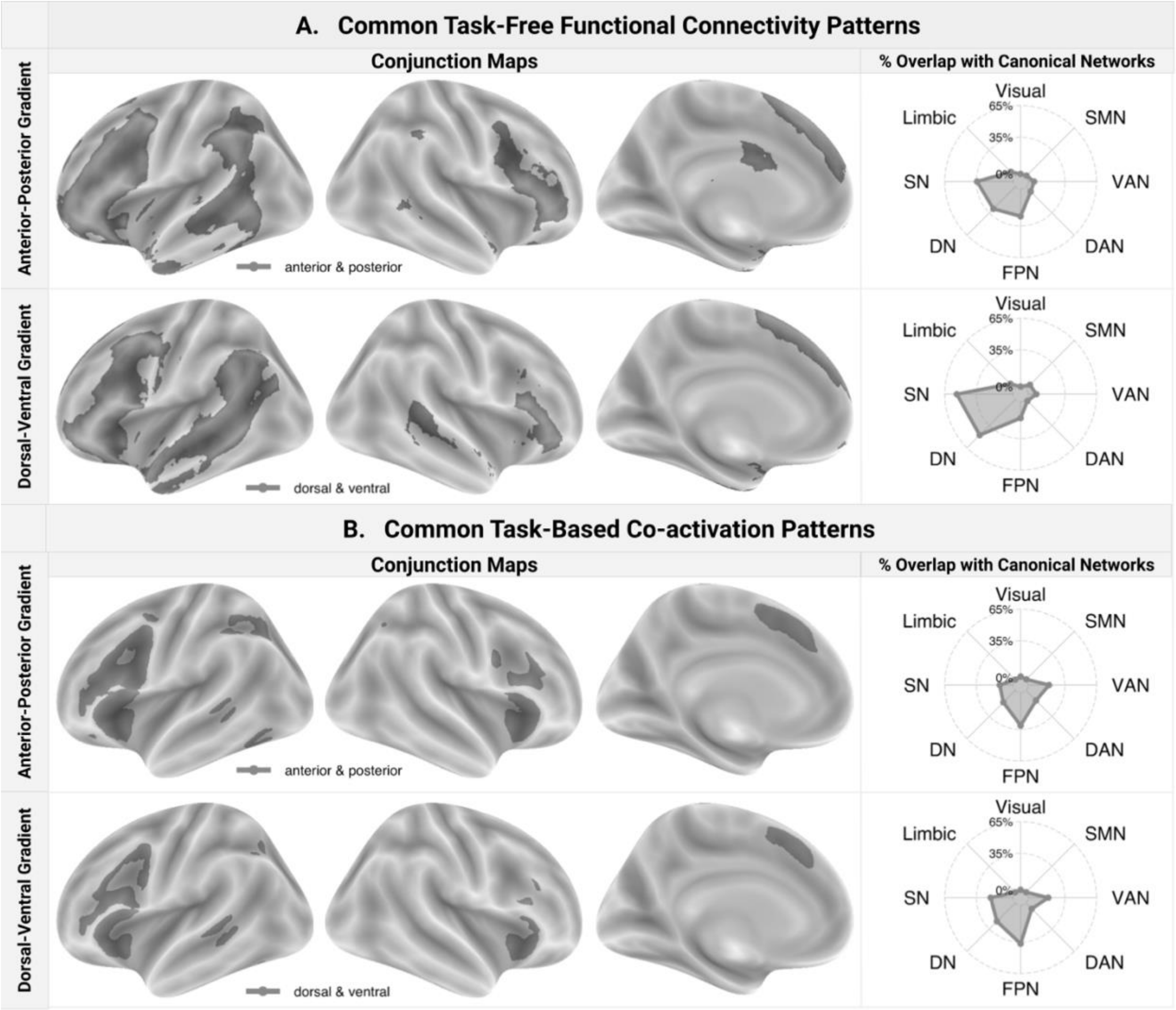

Figure S8. Conjunction maps showing common regions of (A) task-free functional connectivity and (B) co-activation between the ‘gradient extremes’ clusters located at the extremes of the respective gradients. The spider plots in the right column show the percentage of overlap between the contrast maps and canonical networks.

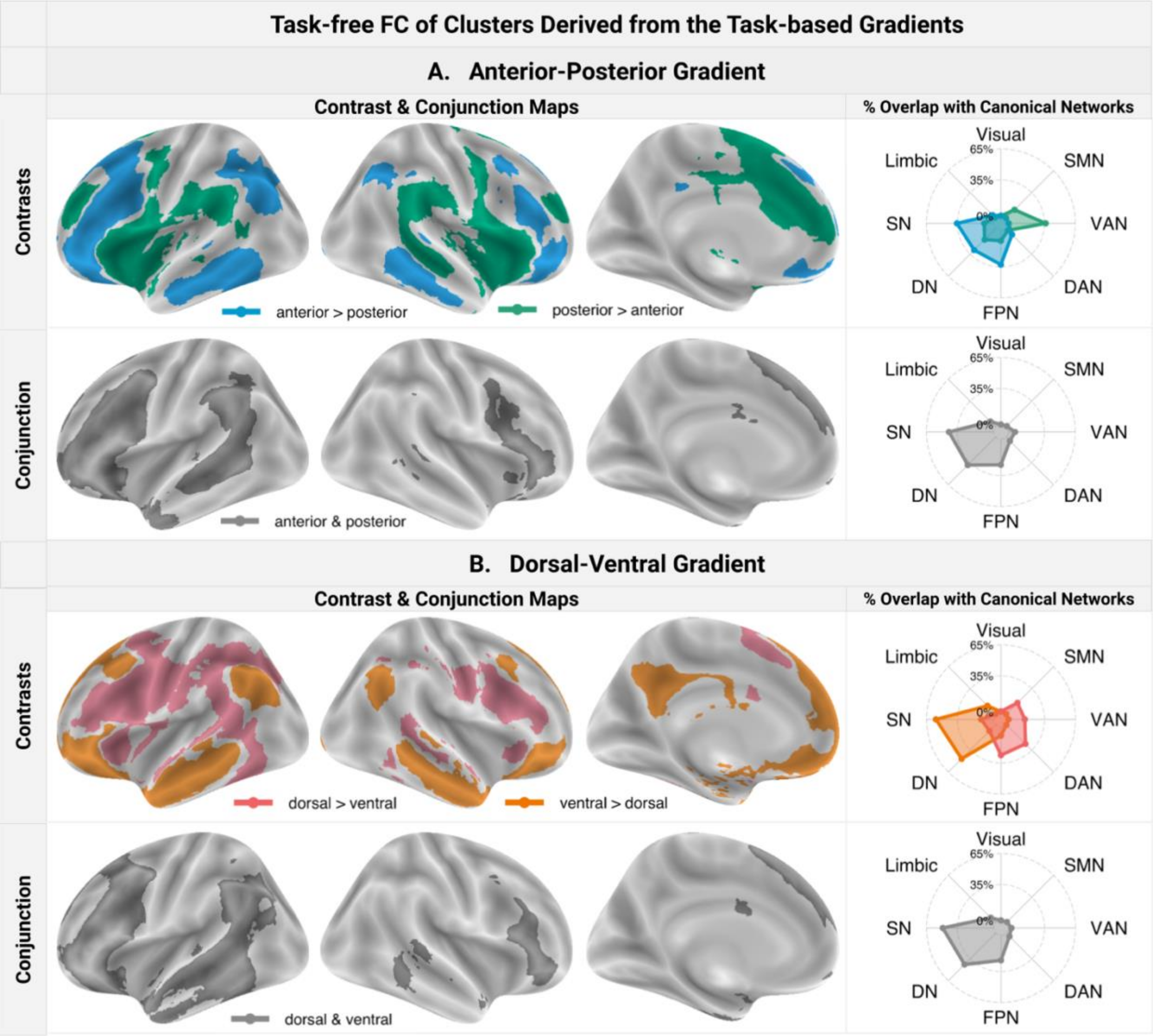

Figure S9. Contrast and conjunction maps showing regions of common and differential functionally coupling at rest between 'gradient extremes' clusters representing the edges of the task-based FC (A) anterior-posterior gradient map and (B) dorsal-ventral gradient map. The spider plots in the right column show the percentage of overlap between the contrast maps and canonical networks.

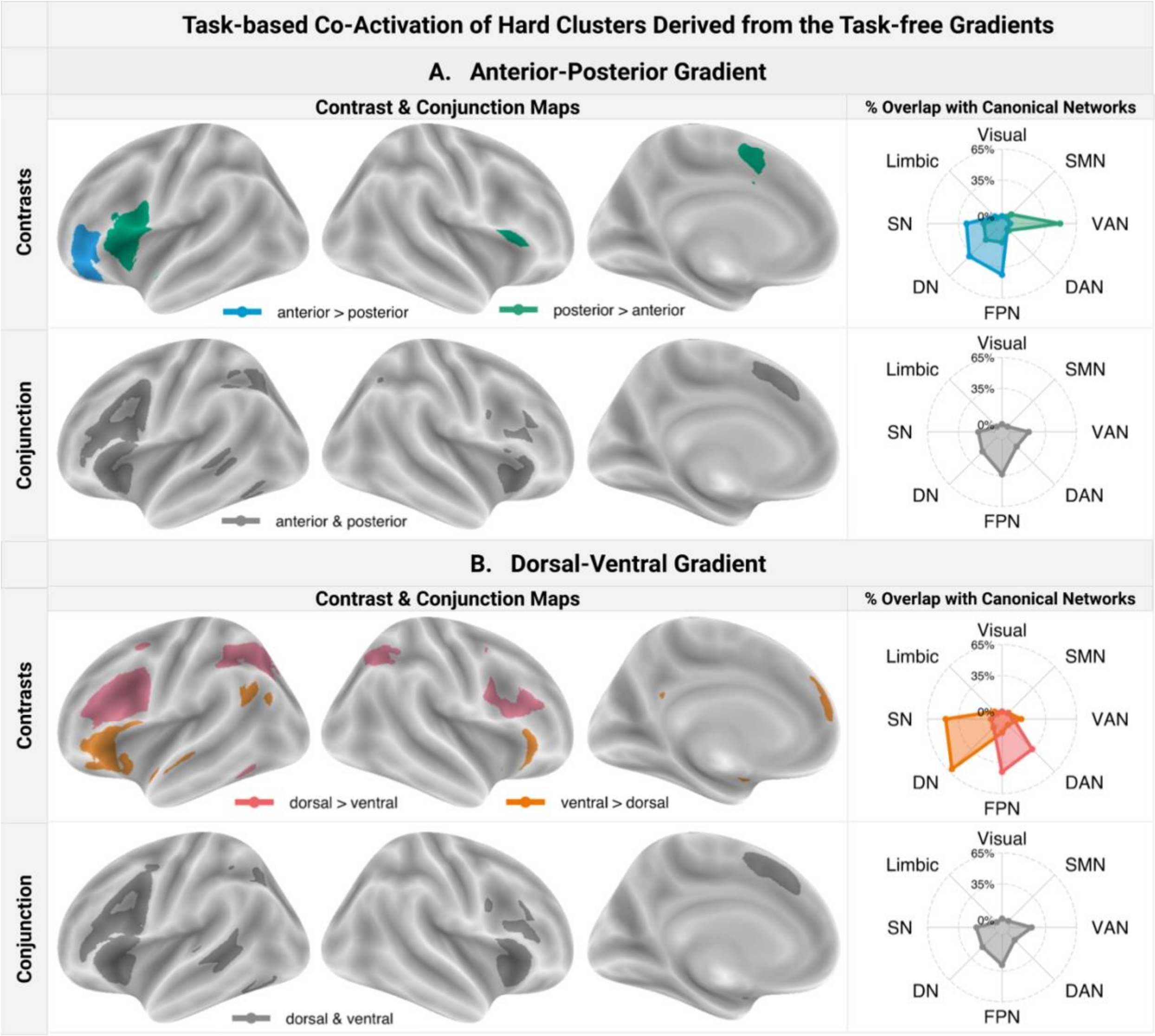

Figure S10. Contrast and conjunction maps showing regions of common and differential task-constrained co-activation across cognitive domains between 'gradient extremes' clusters representing the edges of the task-free FC (A) anterior-posterior gradient map and (B) dorsal-ventral gradient map. The spider plots in the right column show the percentage of overlap between the contrast maps and canonical networks.

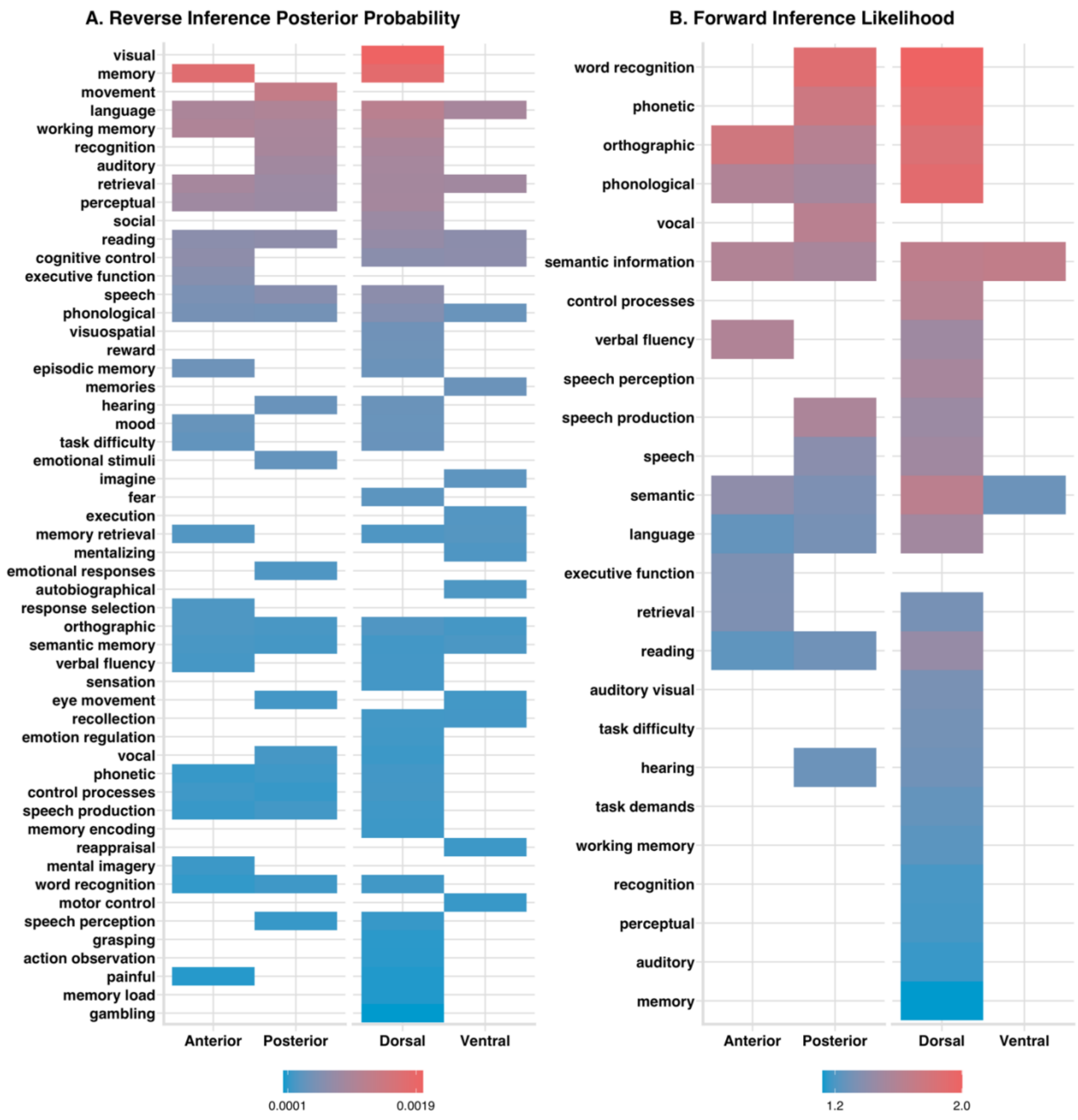

Figure S11. Functional terms associated with the IFG clusters derived based on the task-constrained gradients according to the A) specificity/reverse inference analyses and B) consistency/forward inference analyses. The colour indicates the effect sizes, with red colours suggesting greater association. Only statistically significant associations are highlighted. Synonymous terms with similar pattern of associations across the LIFG clusters were excluded.

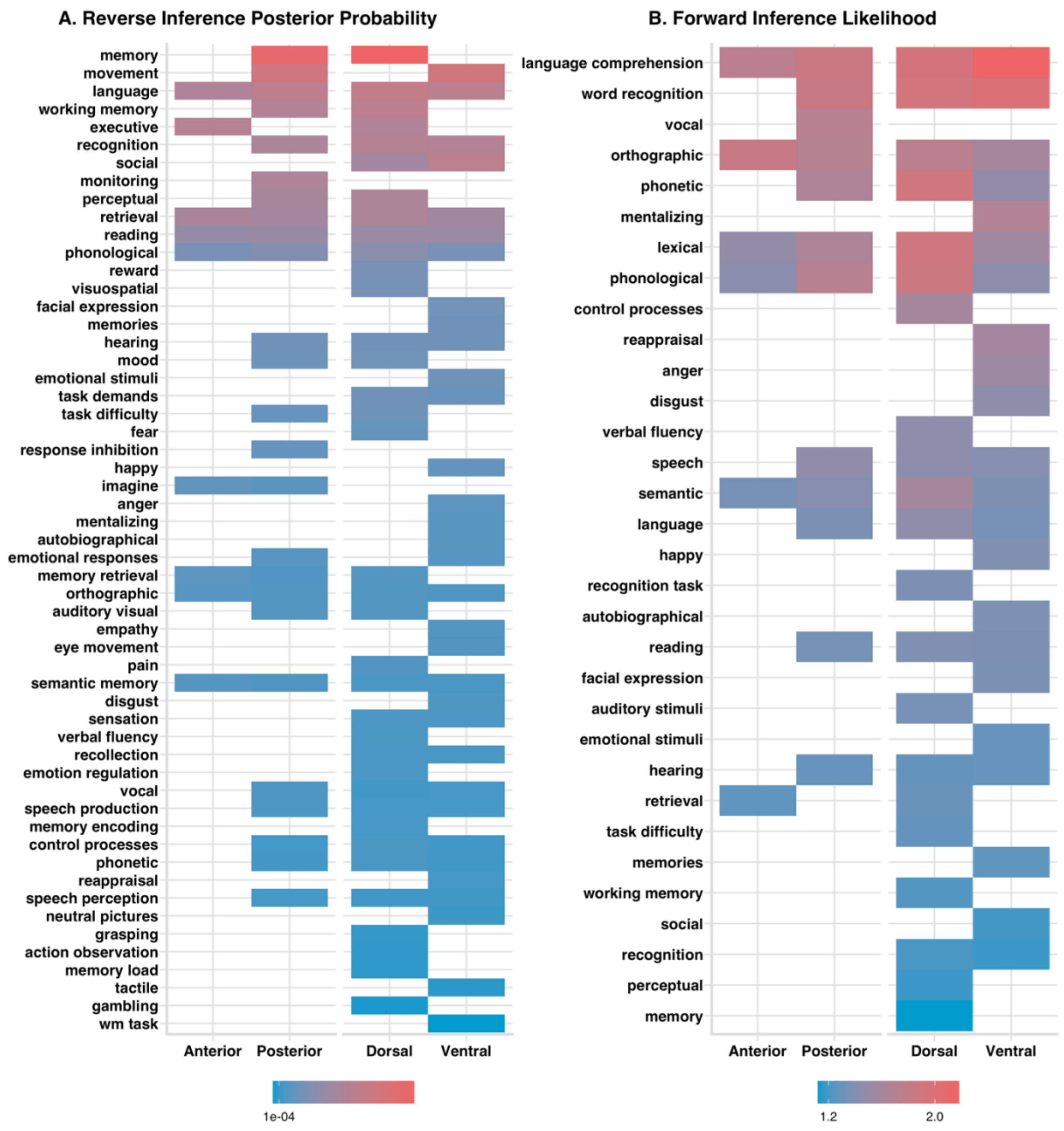

Figure S12. Functional terms associated with the IFG clusters derived based on the task-free gradients according to the A) specificity/reverse inference analyses and B) consistency/forward inference analyses. The colour indicates the effect sizes, with red colours suggesting greater association. Only statistically significant associations are highlighted. Synonymous terms with similar pattern of associations across the LIFG clusters were excluded.

Table S1. The MNI coordinates for the center of gravity of the ‘gradient extremes’ clusters representing the edges of the task-free and task-based gradient maps.

Cluster	Task-free Gradients			Task-based Gradients		
	X	Y	Z	X	Y	Z
Anterior	-44	40	-6	-48	39	1
Posterior	-50	17	9	-44	21	2
Dorsal	-50	23	21	-51	21	22
Ventral	-44	27	-5	-44	38	-11

Table S2. The number of studies from the NeuroQuery database that reported at least one activation coordinate in each ‘gradient extreme’ cluster. These studies were used as the input to MACM and functional decoding analyses.

Cluster	Task-free Gradients	Task-based Gradients
Anterior	664	851
Posterior	1064	1164
Dorsal	1332	1298
Ventral	1098	627

Table S3. Results of the seed-based resting-state functional connectivity analyses conducted on 'gradient extremes' clusters extracted from the **anterior-posterior task-free gradient**.

Analysis	AAL Label	Cluster Size (mm^3)	Max Z Value	X	Y	Z
Anterior Cluster > Posterior Cluster	Frontal_Inf_Orb_L	51,200	21	-36	38	-12
	Angular_L	18,712	18	-44	-68	44
	Frontal_Inf_Orb_R	6,832	17	38	38	-12
	Cerebelum_Crus2_R	17,824	16	46	-72	-40
	Temporal_Mid_L	16,336	16	-64	-44	-10
	Angular_R	6,848	15	40	-72	46
	Cerebelum_Crus2_L	10,336	14	-42	-72	-40
	Temporal_Mid_R	6,568	14	62	-40	-8
	Rectus_R	4,216	14	4	44	-16
	Frontal_Inf_Tri_L	912	13	-52	24	28
	Frontal_Mid_R	2,952	11	32	20	50
	Cerebelum_9_R	520	11	2	-56	-50
	Precentral_L	672	11	-46	8	36
	Frontal_Inf_Tri_R	2,320	9	52	34	22
	Vermis_10	512	8	2	-48	-34
	Temporal_Pole_Mid_L	1,000	7	-32	8	-38
Posterior Cluster > Anterior Cluster	Frontal_Inf_Oper_L	17,720	20	-52	16	2
	Cingulum_Mid_L	35,744	19	-6	14	38
	Frontal_Inf_Orb_R	17,056	18	50	18	-4
	SupraMarginal_L	21,256	16	-56	-40	26
	SupraMarginal_R	14,000	16	58	-30	32
	Precentral_R	2,032	15	54	6	42
	Frontal_Mid_L	5,776	14	-30	50	24
	Frontal_Mid_R	2,760	14	34	46	30
Anterior Cluster ∩ Posterior Cluster	Frontal_Inf_Tri_L	26,192	22	-54	20	20
	Frontal_Inf_Tri_L	5,216	22	-54	22	4
	Supp_Motor_Area_L	23,232	19	-2	22	60
	Parietal_Inf_L	32,240	18	-54	-44	48
	Frontal_Inf_Tri_R	4,320	14	48	40	0
	Frontal_Inf_Orb_R	1,120	14	56	30	-2
	Frontal_Inf_Tri_R	1,704	13	58	26	18

	Cerebelum_Crus1_R	832	11	14	-76	-30
	Temporal_Inf_L	2,424	9	-44	-2	-44
	Temporal_Inf_L	856	8	-50	-6	-40
	Fusiform_L	608	8	-44	-40	-18

Table S4. Results of the seed-based resting-state functional connectivity analyses conducted on 'gradient extremes' clusters extracted from the **dorsal-ventral task-free gradient**.

Analysis	AAL Label	Cluster Size (mm^3)	Max Z Value	X	Y	Z
Dorsal Cluster > Ventral Cluster	Frontal_Inf_Tri_L	24,592	19	-44	32	18
	Parietal_Sup_L	26,328	18	-26	-70	46
	Precentral_L	968	16	-44	2	22
	Temporal_Inf_L	14,032	16	-54	-60	-14
	Frontal_Inf_Tri_R	10,872	15	50	38	18
	Frontal_Mid_L	5,280	13	-28	10	66
	Temporal_Inf_R	3,280	13	60	-50	-8
	Cerebelum_8_R	3,968	12	28	-70	-46
	Cerebelum_Crus1_R	832	10	6	-80	-24
	Insula_L	624	8	-42	-2	6
Ventral Cluster > Dorsal Cluster	Frontal_Inf_Tri_L	14,984	19	-42	26	0
	Frontal_Sup_Medial_L	58,464	16	-4	52	16
	Insula_R	12,960	14	32	20	-14
	Temporal_Inf_L	13,808	14	-48	2	-36
	Temporal_Mid_L	10,728	13	-58	-18	-10
	Temporal_Inf_R	9,960	13	48	2	-32
	Cerebelum_Crus1_R	2,232	12	28	-78	-32
	Temporal_Mid_R	4,344	12	54	-28	-8
	Angular_L	12,208	11	-54	-60	30
	Precuneus_L	1,576	11	-12	-52	32
	Temporal_Sup_R	712	10	58	-44	24
	Cerebelum_Crus2_R	648	9	24	-88	-38
Dorsal Cluster ∩	Frontal_Inf_Tri_L	85,432	24	-54	22	18
	Frontal_Sup_Medial_L	21,624	17	-2	38	46

Ventral Cluster	Frontal_Inf_Tri_R	6,032	14	56	28	20
	Cerebelum_Crus1_R	7,016	14	14	-80	-30
	Temporal_Mid_R	5,424	8	54	-38	8
	Fusiform_L	5,272	8	-30	2	-44

Table S5. Results of the meta-analytic co-activation analyses conducted on 'gradient extremes' clusters extracted from the **anterior-posterior task-based gradient**.

Analysis	AAL Label	Cluster Size (mm^3)	Max Z Value	X	Y	Z
Anterior Cluster > Posterior Cluster	Frontal_Inf_Tri_R	3,208	NA	48	36	18
	Occipital_Mid_R	5,432	4	34	-68	34
	Parietal_Inf_L	9,816	4	-38	-44	42
	Frontal_Inf_Tri_L	20,392	NA	-46	34	12
Posterior Cluster > Anterior Cluster	Insula_R	8,200	NA	44	18	-2
	Insula_L	22,616	NA	-42	18	0
Anterior Cluster ∩ Posterior Cluster	Frontal_Inf_Oper_R	18,200	4	44	20	10
	Supp_Motor_Area_L	15,224	NA	-2	18	46
	Pallidum_L	1,648	NA	-14	6	4
	Parietal_Inf_L	6,544	NA	-36	-54	44
	Frontal_Inf_Oper_L	41,856	NA	-42	18	14
	Occipital_Inf_L	2,312	NA	-44	-60	-12
	Temporal_Mid_L	1,208	NA	-56	-40	0

Table S6. Results of the meta-analytic co-activation analyses conducted on 'gradient extremes' clusters extracted from the **dorsal-ventral task-based gradient**.

Analysis	AAL Label	Cluster Size (mm^3)	Max Z Value	X	Y	Z
Dorsal Cluster > Ventral Cluster	Rolandic_Oper_R	11,904	4	44	4	20
	Angular_R	2,408	4	32	-56	48
	Supp_Motor_Area_L	4,976	NA	-2	12	48
	Parietal_Sup_L	8,552	4	-24	-62	50

	Frontal_Inf_Oper_L	28,584	NA	-46	16	22
	Fusiform_L	520	3	-42	-58	-18
Ventral Cluster > Dorsal Cluster	Frontal_Inf_Orb_R	4,048	4	36	22	-18
	Frontal_Sup_Medial_L	1,080	4	-6	42	40
	Frontal_Inf_Orb_L	17,152	NA	-40	34	-10
	Angular_L	2,720	NA	-48	-62	30
Dorsal Cluster ∩ Ventral Cluster	Insula_R	9,136	NA	40	22	-4
	Supp_Motor_Area_L	9,800	NA	-2	20	46
	Frontal_Inf_Tri_L	30,888	NA	-44	20	12
	Parietal_Inf_L	2,696	NA	-36	-56	46
	Temporal_Mid_L	2,824	NA	-56	-38	0

References

Cerliani, L., Thomas, R. M., Jbabdi, S., Siero, J. C. W., Nanetti, L., Crippa, A., Gazzola, V., D’Arceuil, H., & Keysers, C. (2012). Probabilistic tractography recovers a rostrocaudal trajectory of connectivity variability in the human insular cortex. *Human Brain Mapping, 33*(9), 2005–2034. https://doi.org/10.1002/hbm.21338

Jackson, R. L., Hoffman, P., Pobric, G., & Lambon Ralph, M. A. (2016). The Semantic Network at Work and Rest: Differential Connectivity of Anterior Temporal Lobe Subregions. *The Journal of Neuroscience, 36*(5), 1490–1501. https://doi.org/10.1523/JNEUROSCI.2999-15.2016

Kong, R., Li, J., Orban, C., Sabuncu, M. R., Liu, H., Schaefer, A., Sun, N., Zuo, X.-N., Holmes, A. J., Eickhoff, S. B., & Yeo, B. T. T. (2019). Spatial Topography of Individual-Specific Cortical Networks Predicts Human Cognition, Personality, and Emotion. *Cerebral Cortex*, *29*(6), 2533–2551. https://doi.org/10.1093/cercor/bhy123

Yeo, B. T., Krienen, F. M., Sepulcre, J., Sabuncu, M. R., Lashkari, D., Hollinshead, M., Roffman, J. L., Smoller, J. W., Zöllei, L., Polimeni, J. R., Fisch, B., Liu, H., & Buckner, R. L. (2011). The organization of the human cerebral cortex estimated by intrinsic functional connectivity. *Journal of Neurophysiology*, *106*(3), 1125–1165. https://doi.org/10.1152/jn.00338.2011
